# Assessing the impact of insulin resistance trajectories on cardiovascular disease risk using longitudinal targeted maximum likelihood estimation

**DOI:** 10.1186/s12933-025-02651-6

**Published:** 2025-03-10

**Authors:** Yaning Feng, Liangying Yin, Haoran Huang, Yongheng Hu, Sitong Lin

**Affiliations:** 1 School of Medical Technology and Information Engineering, Zhejiang Chinese Medical University, Hangzhou, China; 2 School of Biomedical Sciences, The Chinese University of Hong Kong, Hong Kong, China

**Keywords:** Cardiovascular disease, Insulin resistance, TyG-BMI, TyG index, Longitudinal study, Targeted maximum likelihood estimation

## Abstract

**Background:**

Cardiovascular disease (CVD) is closely associated with Insulin Resistance (IR). However, there is limited research on the relationship between trajectories of IR and CVD incidence, considering both time-invariant and time-varying confounders. We employed advanced causal inference methods to evaluate the longitudinal impact of IR trajectories on CVD risk.

**Methods:**

The data for this study were extracted from a Chinese nationwide cohort, named China Health and Retirement Longitudinal Study (CHARLS). Triglyceride-glucose (TyG) index and TyG body mass index (BMI) were used as surrogate markers for IR, and their changes were recorded as exposures. Longitudinal targeted maximum likelihood estimation (LTMLE) was used to study how dynamic shifts in IR trajectories (i.e., increase, decrease, etc.) influence long-term CVD risk, adjusting for both time-invariant and time-varying confounders.

**Results:**

A total of 3,966 participants were included in the analysis, with 2,152 (54.3%) being female. The average age at baseline was 58.28 years. Over the course of a 7-year follow-up period, 499 (12.6%) participants developed CVD. Four distinct trajectories of TyG index and TyG-BMI were identified: low stable, increasing, decreasing, and high stable. LTMLE analyses revealed individuals in the ‘high stable’ and ‘increasing’ groups had a significantly higher risk of developing CVD compared to those in the ‘low stable’ group, while the ‘decreasing’ group showed no significant differences. Specifically, when the exposure was set as TyG-BMI, the odds of CVD in the ‘high stable’ group were 1.694 (95% CI: 1.361–2.108) times higher than in the ‘low stable’ group. Similar trends were observed across other models, with ORs of 1.708 (95% CI: 1.367–2.134) in Model 2, 1.389 (1.083–1.782) in Model 3, 1.675 (1.185–2.366) in Model 4, and 1.375 (95% CI:1.07 − 1.768) in Model 5. When the exposure was changed to the TyG index, the results remained consistent, with a slightly lower magnitude of the odds ratios.

**Conclusions:**

High stable and increasing TyG-BMI and TyG index trajectories were associated with the risk of CVD. TyG-BMI consistently exhibited higher odds ratios (ORs) of CVD risk when comparing with TyG index. Early identification of IR trajectories could provide insights for preventing CVD later in life.

**Supplementary Information:**

The online version contains supplementary material available at 10.1186/s12933-025-02651-6.

## Introduction

Cardiovascular disease (CVD) remains the leading cause of death worldwide, contributing to 34.9% of all global fatalities in 2022 [1]. In China, the number of deaths attributed to CVD increased significantly from 3.1 million in 2005 to 4.6 million in 2020, and the burden of premature mortality due to CVD has remained persistently high [2]. Insulin resistance (IR) is a key factor in the development and progression of CVD [3]. Although the hyperinsulinemic-euglycemic clamp is considered the gold standard for measuring IR, its complexity and invasive nature limit its practical use in clinical settings. Recently, the triglyceride-glucose (TyG) index has emerged as a simpler, noninvasive alternative for assessing IR [4]. Studies indicate that the TyG index may be more effective than the homeostatic model assessment for insulin resistance (HOMA-IR) in predicting cardiovascular and metabolic outcomes. This is because the TyG index encompasses both lipid and glucose metabolism, providing a more comprehensive view of metabolic health [5–8]. However, while the TyG index is a valuable marker for IR, it may underestimate metabolic risks in individuals with significant obesity who maintain normal triglyceride and glucose levels. Similarly, body mass index (BMI) alone does not account for variations in insulin sensitivity among individuals with comparable BMI values. By combining these two measures, the TyG-BMI enhances the ability to identify individuals at higher risk for metabolic disorders, providing a more comprehensive evaluation of metabolic health than the TyG index alone [9, 10].

Many current studies have depended on single-time-point assessments of insulin resistance (IR), which do not account for its fluctuations over time. Similar to the way blood pressure patterns evolve throughout life [11, 12], IR can increase, remain stable, or decrease over time. Tracking these temporal trends in IR may provide valuable insights into CVD risk, enabling improved risk stratification and facilitating more targeted interventions. While some research has linked increasing HOMA-IR trajectories to heightened CVD risk and mortality [13], and others have examined changes in the TyG index without accounting for time-varying confounders [14], these approaches often overlook significant temporal fluctuations in IR. The association between long-term TyG index trajectories and CVD outcomes, particularly when adjusting for time-varying confounders, remains inadequately investigated.

Accounting for time-varying confounders is critical when estimating the causal effects of IR on CVD. Such confounders include other comorbidities, cardiometabolic biomarkers (e.g., HDL and LDL), and inflammatory markers. However, traditional analytical methods [15], such as time-dependent Cox regression, random-effects models, and generalized estimating equations, are prone to bias in the presence of time-varying confounders [16]. Addressing these biases necessitates the application of more advanced statistical methodologies [17].

Targeted maximum likelihood estimation (TMLE) [17] is a doubly robust method designed to estimate causal effects with increased precision. Its extension, longitudinal TMLE (LTMLE) [18], adapts TMLE principles to the complexities of longitudinal studies, such as time-varying treatments and confounders. The LTMLE framework offers several advantages for examining insulin resistance (IR) trajectories:

1. Handling time-varying exposure. LTMLE can effectively manage time-varying exposures, which traditional methods often fail to address. This is particularly relevant for studying how temporal changes in IR influence CVD. For instance, across two follow-up time points, a subject could have persistently elevated IR (1,1), elevated IR at only the first time point (1,0), or elevated IR at only the second time point (0,1). LTMLE accommodates these dynamic changes, enabling a nuanced analysis of their effects on CVD risk.
2. Addressing time-varying confounders. The framework is designed to handle time-varying covariates, such as cardiometabolic biomarkers. This capability positions LTMLE as a robust tool for causal inference in longitudinal studies, overcoming the limitations of conventional methods like Cox regression and linear mixed models [19], which struggle to adequately account for time-varying confounders.
3. Double Robustness. LTMLE is doubly robust, meaning it yields consistent estimates if either the outcome model or the treatment mechanism is correctly specified. This property distinguishes LTMLE from approaches like mixed models, which typically rely on the correct specification of the outcome model. Double robustness enhances the reliability and accuracy of causal effect estimation, even under model misspecification.

Given these strengths, we utilized the LTMLE framework [20] to investigate the impact of longitudinal IR trajectories on CVD. LTMLE was specifically designed to address challenges in longitudinal studies where both exposures and confounders vary over time. In our study of IR trajectories, this is particularly relevant as IR status can both influence and be influenced by factors like lifestyle changes, and concurrent health conditions over time. Previous simulation studies have demonstrated LTMLE’s superior performance in such scenarios. For example, Petersen et al. (2014) [21] showed that LTMLE provides consistent estimates even in the presence of strong time-varying confounding, where traditional methods may yield biased results. Their simulation studies demonstrated that LTMLE reduced bias by 25–50% compared to conventional approaches when analyzing longitudinal data with time-varying confounders.

We incorporated the TyG index and TyG-BMI as the main surrogate markers of insulin resistance, and other four additional markers, including metabolic score for insulin resistance (MetS-IR), single-point insulin sensitivity estimator (SPISE), triglycerides to HDL-C ratio (TG/HDL-C), and TyG waist circumference-to-height ratio (TyG-WHtR).

In summary, this study addressed the following objectives:

1. To explore how dynamic shifts in IR trajectories (i.e., increase, decrease, stable) influence long-term CVD.
2. Estimate the causal effects of IR trajectories on CVD risk while accounting for time-varying confounders, using a doubly robust framework in a nationwide Chinese cohort.

By employing LTMLE, our study provides a more comprehensive and accurate evaluation of the long-term effects of IR trajectories on CVD. The approach integrates complex exposure patterns and time-varying confounders, offering insights applicable to real-world settings.

## Methods

### Data source and study population

The data for this study were obtained from the China Health and Retirement Longitudinal Study (CHARLS), a nationally representative longitudinal cohort focusing on individuals aged 45 years and older. The CHARLS baseline survey, conducted in 2011 (Wave 1), included 17,708 participants from 10,257 households across 150 counties/districts and 450 villages or residential committees throughout China. Follow-up surveys were conducted biennially in 2013, 2015, 2018, and 2020, corresponding to Wave 2, Wave 3, Wave 4, and Wave 5, respectively. Blood samples were collected during Wave 1 and Wave 3, with 11,847 and 13,420 participants providing samples in those waves. Detailed descriptions of the sampling methods, anthropometric measurements, and blood biomarker collection have been published previously [22, 23].

**Fig. 1:**
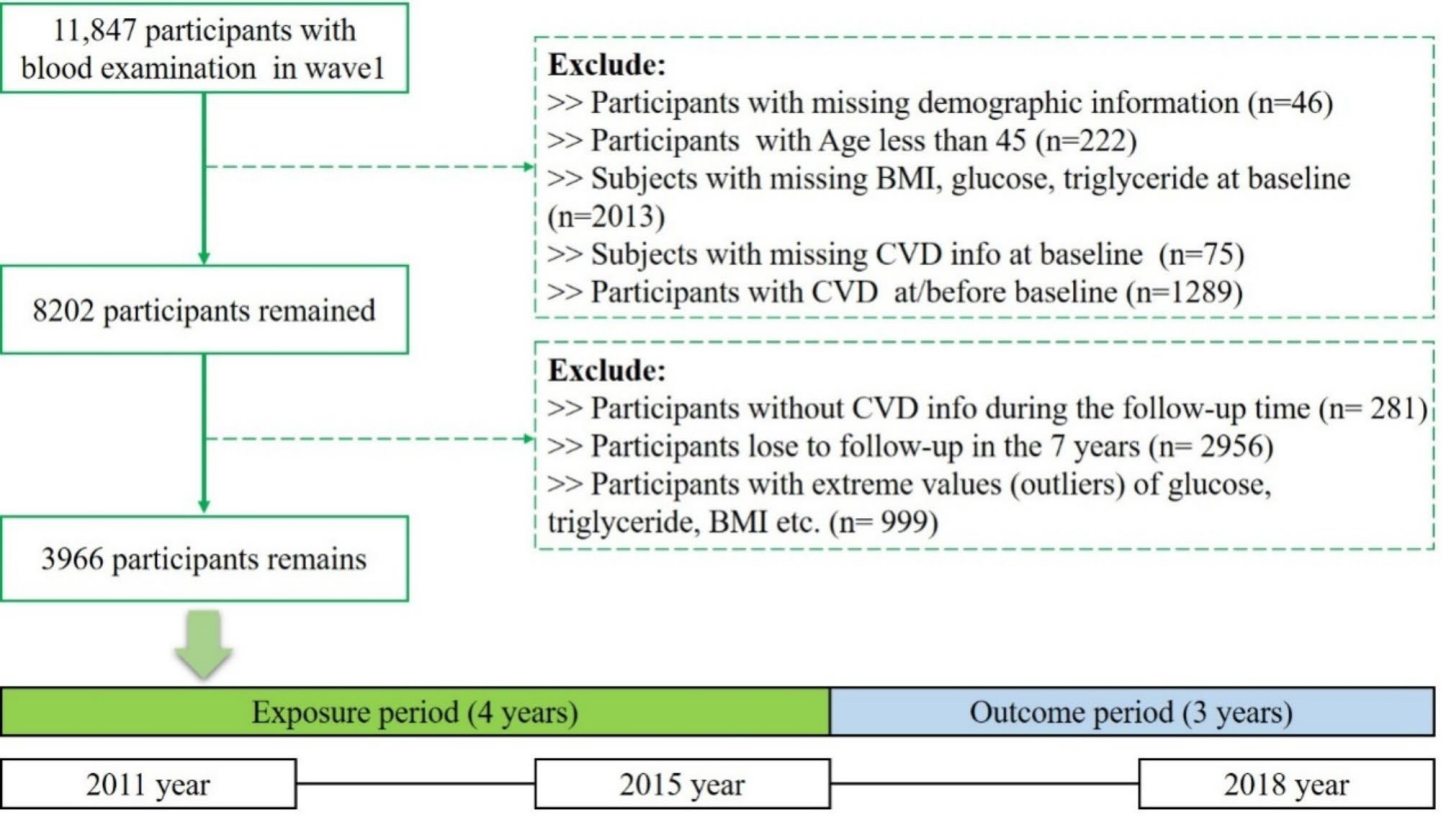
Flowchart for the selection of participants in the cohort study (CHARLS, 2011–2018). Abbreviations: CHARLS, China Health and Retirement Longitudinal Study; BMI, body mass index; CVD, cardiovascular disease.

This study did not conduct a formal a priori sample size calculation due to its observational nature and the secondary use of data from the CHARLS cohort. Given the longitudinal design, the sample size was determined based on available data after rigorous application of eligibility criteria to ensure accurate and reliable analyses. We initially included 11,847 participants who had blood samples collected at Wave 1. Participants with missing demographic data (*n* = 46) or incomplete information on BMI, triglycerides (TG), or fasting blood glucose (FBG) at Wave 1 (*n* = 2,013) were excluded from the analysis. Participants younger than 45 years (*n* = 222) or those lacking CVD data at Wave 1 (*n* = 75) were also excluded. Additionally, we excluded individuals with a baseline diagnosis of CVD (*n* = 1,289) and those who either did not develop CVD during follow-up or were lost to follow-up. Extreme outliers, defined as values exceeding three standard deviations from the mean for glucose, TG, or BMI, were removed. After applying these criteria, a total of 3,966 eligible participants remained for analysis (Fig. 1).

The CHARLS study was carried out following the guidelines outlined in the Declaration of Helsinki and was approved by the Institutional Review Board of Peking University (IRB00001052-11015). Written informed consent was obtained from all participants before enrollment. The study adhered to the Strengthening the Reporting of Observational Studies in Epidemiology (STROBE) guidelines for reporting observational research [24].

### Exposure

In this study, two main surrogate markers were used to assess insulin resistance (IR): the TyG index and the TyG-BMI. Four additional IR markers were used as part of the sensitivity analysis, including metabolic score for insulin resistance (MetS-IR), single-point insulin sensitivity estimator (SPISE), triglycerides to HDL-C ratio (TG/HDL-C), TyG waist circumference-to-height ratio (TyG-WHtR) [25–31]. The TyG index [4] was calculated, using the following formula: TyG index = ln [triglyceride (mg/dL) × glucose (mg/dL) ÷ 2]. BMI was computed as weight (kg) divided by height squared (m²), and the TyG-BMI [32] was calculated, using the following formula: TyG-BMI = ln [triglyceride (mg/dL) × glucose (mg/dL) ÷ 2]× BMI. The definition of other four IR markers are shown in Appendix E. Two time points of exposure were defined in our study. Wave 1 (in 2011 year) was defined as the first time point $\:{t}_{1}$, and also wave 1 was defined as baseline. Wave 3 (in 2015 year) was defined as the second time point $\:{t}_{2}$. $\:{A}_{i}$ refers to the exposure at $\:{t}_{i}$, for example, $\:{A}_{1}$ refers to the exposure at $\:{t}_{1}$. Further details on the trajectory modeling approach are provided in the statistical analysis section.

### Ascertainment of CVD events

The study outcome was the incidence of CVD events. Consistent with previous research [25, 26], incident CVD events were evaluated using a set of standardized questions: “Have you been told by a doctor that you have been diagnosed with a heart attack, coronary heart disease, angina, congestive heart failure, or other heart problems?” or “Have you been told by a doctor that you have been diagnosed with a stroke?” Participants who reported a diagnosis of heart disease or stroke during the follow-up period were classified as having incident CVD.

More specifically, subjects who had CVD in 2011 were excluded, and if the patient was diagnosed with CVD after that until the follow-up period in 2018, he or she was included in the study under our definition of a patient having incident CVD. In this case, the outcome was defined as 1, otherwise it was defined as 0.

### Covariates

This study incorporated both time-invariant and time-varying covariates. Time-invariant covariates, measured at baseline (Wave 1), included sex, education level, and residence location. Time-varying covariates were collected at both Wave 1 and Wave 3 and included lifestyle factors (e.g., smoking status and alcohol consumption), comorbidities (e.g., dyslipidemia, hypertension, diabetes, kidney and liver disease), and laboratory parameters (e.g., HDL, LDL, and hsCRP levels). Marital status was mainly included as a time-invariant covariate, but also incorporated as a time-varying covariate for sensitivity analysis.

### Statistical analysis

Continuous variables were described using means and standard deviations (SD), whereas categorical variables were reported as frequencies and percentages. The study included data from three time points: Wave 1 (2011), Wave 3 (2015), and Wave 4 (2018). The exposure period was defined as spanning from 2011 to 2015, while the outcome period covered 2015 to 2018 (see Fig. 1). The dataset’s characteristics were analyzed by outcome status (presence or absence of CVD events) and by levels of insulin resistance (IR), represented by the TyG index and TyG-BMI. Given the absence of clinically established cutoff points for the TyG index, we followed previous studies [33, 34] and used the median value of TyG as a threshold, with values above the median categorized as elevated. Specifically, exposure was coded as 1 for elevated TyG levels and 0 otherwise. The exposure at each time point, $\:{A}_{i}$​, was assigned a value of 1 if TyG levels were elevated at $\:{t}_{i}$and 0 otherwise. Trajectories of the exposure were classified into four groups: low-stable, increasing, decreasing, and high-stable, as detailed in Table S1.

The missing data rates for the covariates are summarized in Table S2, ranging from 0.00014 to 0.01884. It was assumed that the missing data were missing at random. To address this, five imputed datasets were generated, and the results were pooled using the multiple imputation by chained equations (MICE) Markov chain Monte Carlo method.

We applied longitudinal targeted maximum likelihood estimation (LTMLE) [20] to investigate the impact of IR trajectories on CVD. LTMLE is a doubly robust method for causal effect estimation [35], integrating both an outcome model and a propensity score (PS)-based treatment model. This approach minimizes bias due to potential model misspecification. The LTMLE process involves three primary steps: (1) Using treatment and confounder variables to estimate the initial expected outcomes; (2) Estimating the propensity score, which represents the probability of receiving treatment given covariates; (3) Updating the initial outcome model using the propensity score. This “doubly robust” methodology ensures consistent estimates as long as either the outcome model or the treatment assignment mechanism is correctly specified. Additional details are provided in Appendix A of the Supplementary Materials.

**Fig. 2:**
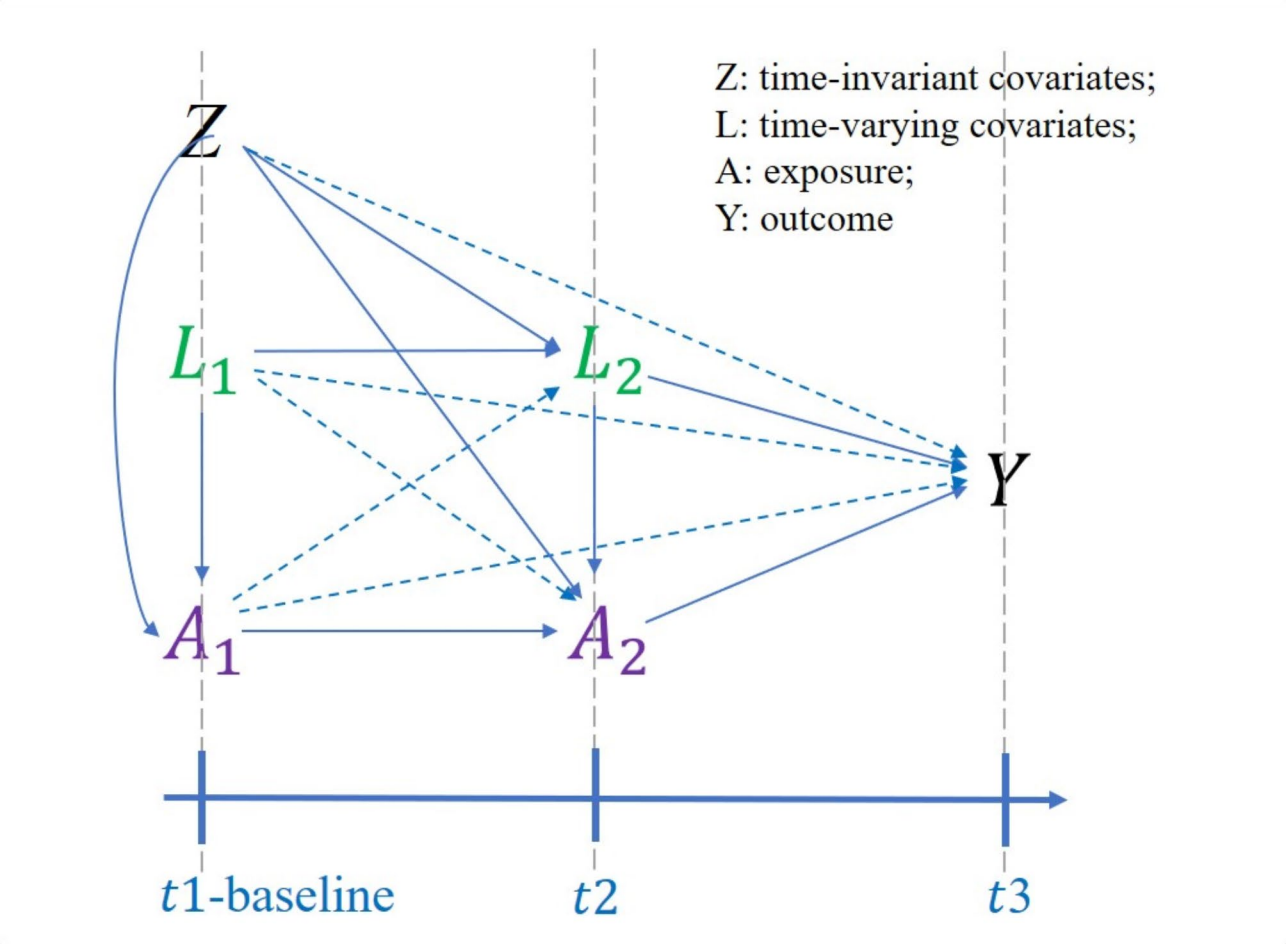
Assumed Directed Acyclic Graph (DAG) illustrating sequential relationships among exposure, outcome, and time-varying confounders across multiple time points. *Z* refers to the baseline covariates; *L* refers to the time-varying covariates; *A* refers to the exposure, including TyG-BMI and TyG index; 3 time points were included, including *t1* (in 2011), *t2* (in 2015), and *t3* (in 2018)

Our analysis incorporated three time points, under the assumption that earlier exposures and confounders influence subsequent exposures and confounders. More specific details regarding these assumptions can be found in Appendix B. The observed data structure for this study is represented as O = {Z,$\:{L}_{1}$, $\:{L}_{2}$, $\:{A}_{1}$, $\:{A}_{2}$, Y}, and the assumed network is depicted in Fig. 2.

We conducted several sensitivity analyses to assess the robustness of our findings: (1) model variations with covariates. We defined five models by incorporating different sets of time-invariant and time-varying covariates. Model 1 was adjusted for age and gender, whereas Model 2 included additional adjustments for residence (hukou), education level, marital status, smoking habits, and alcohol consumption. In Model 3, we further included comorbidities such as hypertension, dyslipidemia, diabetes, and kidney disease, based on Model 2. In addition, Model 4 adjusted for laboratory parameters, including HDL, LDL, and hsCRP, in addition to the covariates in Model 2. Model 5 was defined to further include liver disease, time-varying marital status etc. Detailed descriptions of these models are provided in Appendix C; (2) Several IR markers were used to assess the robustness of our findings from various perspectives. These markers included the TyG index, TyG-BMI, MetS-IR, SPISE, TG/HDL-C, and TyG-WHtR. (3) comparison of estimation methods. we compared the results of the LTMLE with those obtained using the G-computation method to evaluate the consistency and robustness of our findings; (4) SuperLearner library variations. We assessed the stability of our results by using multiple SuperLearner libraries, comprising a different combination of models, including generalized linear model (GLM), random forest, and xgboost etc. (see Appendix D). (5) E-Value for unmeasured confounding. We used the E-value to evaluate the strength of potential unmeasured confounders required to nullify the observed associations, providing insight into the robustness of our causal inferences in the context of observational data.

All statistical analyses were conducted using R (version 4.4.1; R Foundation for Statistical Computing, Vienna, Austria). Multiple imputation was performed using the ‘mice’ package (version 3.16.0) in R. Longitudinal targeted maximum likelihood estimation and G-computation were conducted via ‘ltmle’ (version 1.3.0) package. E-value was calculated by ‘EValue’ (version 4.1.3) package in R. A two-sided P value of < 0.05 was statistically significant.

## Results

### Clinical characteristics of the study population

The study involved 3,966 participants, with a mean age of 58.28 years (SD = 8.53). Of these, 2,152 (54.3%) were female at baseline. A total of 499 participants were diagnosed with CVD during the study. Exposure data were collected at two time points, in 2011 and 2015, while outcomes were assessed in 2018. Table 1 provides a summary of the participant characteristics. At baseline, the mean TyG index was 8.57 (SD = 0.54), which slightly increased to 8.61 (SD = 0.54) at the second time point. The TyG-BMI showed a similar trend, with a mean of 199.77 (SD = 35.24) at the baseline, rising to 203.23 (SD = 36.30) at the second measurement. Continuous exposure data were dichotomized using the median as the cutoff. At baseline, 48.6% of participants had elevated TyG index values, which increased to 50.1% by the second time point. Similarly, the percentage of participants with elevated TyG-BMI increased from 46.6% at baseline to 52.1% at follow-up. Participants with CVD were more likely to exhibit higher TyG index and TyG-BMI values at both time points compared to those without CVD. Additional details regarding the time-invariant and time-varying covariates are outlined in Table 1.

**Table 1:** Characteristics of study population by CVD status at follow-up

Attributes	Characteristics	Time points	Total (*n* = 3966)	Non-CVD (*n* = 3467)	CVD (*n* = 499)	*P*
Binary exposure	TyG index = High (%)	Time point 1	1926(48.6)	1663(48.0)	263(52.7)	0.053
		Time point 2	1986(50.1)	1700(49.0)	286(57.3)	0.001
	TyG-BMI = High (%)	Time point 1	1847(46.6)	1578(45.5)	269(53.9)	0.001
		Time point 2	2065(52.1)	1764(50.9)	301(60.3)	< 0.001
Continuous exposure	TyG index (mean (SD))	Time point 1	8.57(0.54)	8.57(0.54)	8.64(0.53)	0.006
		Time point 2	8.61(0.54)	8.60(0.54)	8.66(0.51)	0.037
	TyG-BMI (mean (SD))	Time point 1	199.77(35.24)	198.91(34.93)	205.75(36.78)	< 0.001
		Time point 2	203.23(36.30)	202.44(36.19)	208.72(36.63)	< 0.001
Time-varying covariates	Glucose mg/dL (mean (SD))	Time point 1	103.26(15.98)	103.01(15.62)	104.97(18.23)	0.011
		Time point 2	97.26(15.67)	97.18(15.60)	97.76(16.09)	0.442
	Cholesterol mg/dL (mean (SD))	Time point 1	191.96(36.47)	191.32(36.27)	196.37(37.55)	0.004
		Time point 2	183.87(34.67)	183.71(34.54)	185.01(35.57)	0.433
	TG mg/dL (mean (SD))	Time point 1	117.50(63.36)	116.93(63.59)	121.51(61.69)	0.131
		Time point 2	128.55(68.29)	128.02(68.58)	132.25(66.14)	0.196
	HDL mg/dL (mean (SD))	Time point 1	52.45(14.88)	52.57(14.84)	51.57(15.17)	0.157
		Time point 2	52.49(12.01)	52.62(12.03)	51.60(11.79)	0.075
	LDL mg/dL (mean (SD))	Time point 1	116.96(32.88)	116.38(32.50)	120.97(35.19)	0.004
		Time point 2	103.33(28.26)	103.13(28.02)	104.65(29.87)	0.262
	hsCRP mg/L (mean (SD))	Time point 1	1.43(1.51)	1.40(1.49)	1.65(1.66)	< 0.001
		Time point 2	2.23(4.75)	2.19(4.46)	2.51(6.41)	0.157
	BMI kg/m2 (mean (SD))	Time point 1	23.24(3.39)	23.16(3.35)	23.77(3.66)	< 0.001
		Time point 2	23.54(3.48)	23.46(3.45)	24.06(3.65)	< 0.001
	SBP mmHg (mean (SD))	Time point 1	128.51(23.72)	127.81(23.29)	133.35(26.01)	< 0.001
		Time point 2	127.96(19.67)	127.10(19.48)	133.92(19.96)	< 0.001
	DBP mmHg (mean (SD))	Time point 1	74.91(11.80)	74.64(11.76)	76.80(11.96)	< 0.001
		Time point 2	75.15(11.51)	74.93(11.63)	76.73(10.57)	0.001
	Hypertension, n (%)	Time point 1	712(18.0)	577(16.6)	135(27.1)	< 0.001
		Time point 2	374(9.4)	304(8.8)	70(14.0)	< 0.001
	Dyslipidemia, n (%)	Time point 1	250(6.3)	189(5.5)	61(12.2)	< 0.001
		Time point 2	156(3.9)	122(3.5)	34(6.8)	0.001
	Diabetes, n (%)	Time point 1	111(2.8)	87(2.5)	24(4.8)	0.006
		Time point 2	76(1.9)	52(1.5)	24(4.8)	< 0.001
	Kidney, n (%)	Time point 1	222(5.6)	186(5.4)	36(7.2)	0.115
		Time point 2	45(1.1)	36(1.0)	9(1.8)	0.199
	Liver, n (%)	Time point 1	135 (3.4)	114 (3.3)	21(4.2)	0.353
		Time point 2	32 (0.8)	25 (0.7)	7 (1.4)	0.186
	Smoking status at t1 (%)	Time point 1				0.005
	Smokers		1226 ( 30.9)	1093 (31.5)	133 (26.7)	
	Non-smokers		2466 (62.2)	2149 (62.0)	317 (63.5)	
	Ex-smokers		274 (6.9)	225 (6.5)	49 (9.8)	
	Smoking status at t2 (%)	Time point 2				0.585
	Smokers		292 (7.4)	258 (7.4)	34 (6.8)	
	Non-smokers		3521 (88.8)	3079 (88.8)	442 (88.6)	
	Ex-smokers		153 (3.9)	130 (3.7)	23 (4.6)	
	Marital status at t1 (%)	Time point 1				0.348
	Married with spouse present		3426 (86.4)	2996 (86.4)	430 (86.2)	
	Married but not living with spouse temporarily for reasons such as work		144 (3.6)	128 (3.7)	16 (3.2)	
	Separated		17 (0.4)	17 (0.5)	0 (0.0)	
	Divorced		19 (0.5)	15 (0.4)	4 (0.8)	
	Widowed		341 (8.6)	293 (8.5)	48 (9.6)	
	Never married		19 (0.5)	18 (0.5)	1 (0.2)	
	Marital status at t2 (%)	Time point 2				0.637
	Married with Spouse Present		3271 (82.5)	2871 (82.8)	400 (80.2)	
	Married but not living with spouse temporarily for reasons such as work		152 (3.8)	134 (3.9)	18 (3.6)	
	Separated		11 (0.3)	10 (0.3)	1 (0.2)	
	Divorced		21 (0.5)	18 (0.5)	3 (0.6)	
	Widowed		490 (12.4)	415 (12.0)	75 (15.0)	
	Never married		19 (0.5)	17 (0.5)	2 (0.4)	
	Cohabitated		2 (0.1)	2 (0.1)	0 (0.0)	
	Drinking status at t1 (%)	Time point 1				0.504
	Drink more than once a month		1020(25.7)	902(26.0)	118(23.6)	
	Drink but less than once a month		338(8.5)	296(8.5)	42(8.4)	
	None of these		2608(65.8)	2269(65.4)	339(67.9)	
	Drinking status at t2 (%)	Time point 2				0.293
	Drink more than once a month		1025(25.8)	910(26.2)	115(23.0)	
	Drink but less than once a month		352(8.9)	304(8.8)	48(9.6)	
	None of these		2589(65.3)	2253(65.0)	336(67.3)	
	Age (mean (SD))	Time point 1	58.28(8.53)	58.02(8.55)	60.07(8.22)	< 0.001
Time-invariant covariates	Gender = Female (%)	Time point 1	2152(54.3)	1859(53.6)	293(58.7)	0.037
	Residence (%)	Time point 1				0.669
	Agricultural		3468(87.4)	3033(87.5)	435(87.2)	
	Non-Agricultural		477(12.0)	417(12.0)	60(12.0)	
	Unified Residence Hukou		21(0.5)	17(0.5)	4(0.8)	
	Education (%)	Time point 1				0.338
	No formal education illiterate		1138(28.7)	977(28.2)	161(32.3)	
	Did not finish primary school but capable of reading or writing		743(18.7)	650(18.7)	93(18.6)	
	Sishu		17(0.4)	16(0.5)	1(0.2)	
	Elementary school		893(22.5)	782(22.6)	111(22.2)	
	Middle school		818(20.6)	729(21.0)	89(17.8)	
	High school		262(6.6)	230(6.6)	32(6.4)	
	Vocational school		52(1.3)	46(1.3)	6(1.2)	
	Two/Three Year College / Associate degree		38(1.0)	34(1.0)	4(0.8)	
	Four Year College / Bachelor’s degree		5(0.1)	3(0.1)	2(0.4)	

Note: 1) time point 1 refers to the data in year 2011 in CHARLS database, while time point 2 refers to the data in year 2015 in CHARLS database

2) Age is a time-varying confounder, and the age at time point 2 can be calculated by the age at time point1 plus 4 years.

3) Abbreviation: TG, Triglycerides; HDL, High-density lipoprotein; LDL, low-density lipoprotein; hsCRP, Immunoturbidimetric assay;

BMI, Body mass index; SBP, Systolic blood pressure; DBP, Diastolic blood pressure; CVD: Cardiovascular disease or stroke

### Latent TyG index and TyG-BMI trajectories

In our study, four distinct trajectories for TyG index and TyG-BMI were defined: low stable, increasing, decreasing, and high stable. The baseline characteristics of each trajectory are presented in Tables 2 and 3. Females were slightly underrepresented in the “low increasing” trajectory. In the “low stable” trajectory, females accounted for 47.8% of the TyG index group and 44.7% of the TyG-BMI group, while the “increasing” and “high stable” trajectories included a higher proportion of females. Furthermore, the mean TyG index showed an increasing trend across the trajectories, ranging from 8.11 (SD = 0.28) in the “low stable” group to 9.07 (SD = 0.37) in the “high stable” group. A similar pattern was observed for TyG-BMI. The levels of triglycerides (TG), BMI, LDL, diabetes, hypertension, dyslipidemia, and kidney disease were higher in the “high stable” trajectory compared to the other three trajectories, whereas HDL levels were lower in the “high stable” group. However, the incidence of kidney disease was similar across all four trajectories. Additional details are provided in Tables 2 and 3.

**Table 2:** Baseline characteristic of the study population in different TyG index trajectories group

Characteristics	Low stable(*n* = 1389)	Increasing(*n* = 651)	Decreasing(*n* = 591)	High stable(*n* = 1335)	P
Glucose mg/dL (mean (SD))	97.98(12.53)	98.42(11.91)	108.93(18.28)	108.61(17.20)	< 0.001
Cholesterol mg/dL (mean (SD))	181.05(32.15)	192.27(35.23)	191.74(36.31)	203.26(37.92)	< 0.001
TG mg/dL (mean (SD))	71.10(19.17)	80.38(19.34)	144.88(52.18)	171.77(63.02)	< 0.001
HDL mg/dL (mean (SD))	58.94(14.90)	55.98(14.26)	49.89(13.74)	45.10(11.74)	< 0.001
LDL mg/dL (mean (SD))	109.68(28.33)	122.47(31.53)	113.86(33.31)	123.21(35.92)	< 0.001
hsCRP mg/L (mean (SD))	1.31(1.51)	1.33(1.39)	1.37(1.49)	1.63(1.57)	< 0.001
Age (mean (SD))	58.79(8.83)	57.22(8.46)	58.76(8.47)	58.06(8.23)	0.001
BMI kg/m2 (mean (SD))	22.07(3.09)	23.15(3.06)	22.97(3.21)	24.60(3.45)	< 0.001
SBP mmHg (mean (SD))	124.28(19.22)	127.92(26.35)	129.62(25.10)	132.71(25.18)	< 0.001
DBP mmHg (mean (SD))	72.67(11.29)	75.17(11.36)	75.03(12.20)	77.07(11.96)	< 0.001
TyG_index (mean (SD))	8.11(0.28)	8.24(0.25)	8.91(0.31)	9.07(0.37)	< 0.001
TyG_BMI (mean (SD))	179.10(26.46)	190.96(26.67)	204.79(30.48)	223.34(33.98)	< 0.001
Gender = Female (%)	664(47.8)	365(56.1)	296(50.1)	827(61.9)	< 0.001
Residence (%)					0.012
Agricultural	1231(88.6)	571(87.7)	531(89.8)	1135(85.0)	
Non-Agricultural	150(10.8)	79(12.1)	55(9.3)	193(14.5)	
Unified Residence Hukou	8(0.6)	1(0.2)	5(0.8)	7(0.5)	
Marital status (%)					0.13
Married with spouse present	1190(85.7)	566(86.9)	494(83.6)	1176(88.1)	
Married but not living with spouse temporarily for reasons such as work	59(4.2)	22(3.4)	22(3.7)	41(3.1)	
Separated	6(0.4)	2(0.3)	3(0.5)	6(0.4)	
Divorced	10(0.7)	1(0.2)	3(0.5)	5(0.4)	
Widowed	112(8.1)	58(8.9)	66(11.2)	105(7.9)	
Never married	12(0.9)	2(0.3)	3(0.5)	2(0.1)	
Education (%)					0.088
No formal education illiterate	370(26.6)	185(28.4)	181(30.6)	402(30.1)	
Did not finish primary school but capable of reading or writing	291(21.0)	114(17.5)	118(20.0)	220(16.5)	
Sishu	10(0.7)	5(0.8)	0(0.0)	2(0.1)	
Elementary school	304(21.9)	158(24.3)	129(21.8)	302(22.6)	
Middle school	287(20.7)	126(19.4)	114(19.3)	291(21.8)	
High school	93(6.7)	48(7.4)	40(6.8)	81(6.1)	
Vocational school	16(1.2)	8(1.2)	4(0.7)	24(1.8)	
Two/Three Year College / Associate degree	15(1.1)	6(0.9)	4(0.7)	13(1.0)	
Four Year College / Bachelor’s degree	3(0.2)	1(0.2)	1(0.2)	0(0.0)	
Hypertension, n (%)	188(13.5)	96(14.7)	98(16.6)	330(24.7)	< 0.001
Dyslipidemia, n (%)	62(4.5)	41(6.3)	30(5.1)	117(8.8)	< 0.001
Diabetes, n (%)	27(1.9)	15(2.3)	16(2.7)	53(4.0)	0.011
Kidney, n (%)	91(6.6)	32(4.9)	31(5.2)	68(5.1)	0.29
Liver, n (%)	60(4.3)	21(3.2)	19(3.2)	35(2.6)	0.104
Smoking status (%)					< 0.001
Smokers	481(34.6)	202(31.0)	209(35.4)	334(25.0)	
Non-smokers	804(57.9)	414(63.6)	340(57.5)	908(68.0)	
Ex-smokers	104(7.5)	35(5.4)	42(7.1)	93(7.0)	
Drinking status (%)					0.001
Drink more than once a month	405(29.2)	167(25.7)	151(25.5)	297(22.2)	
Drink but less than once a month	128(9.2)	46(7.1)	58(9.8)	106(7.9)	
None of these	856(61.6)	438(67.3)	382(64.6)	932(69.8)	

Abbreviations: TG, Triglycerides; TyG, Triglyceride glucose; SBP, Systolic blood pressure; DBP, Diastolic blood pressure; HDL, High-density lipoprotein; LDL, low-density lipoprotein; hsCRP, Immunoturbidimetric assay; BMI, Body mass index

**Table 3:** Baseline characteristic of the study population in different TyG-BMI trajectories group

Characteristics	Low stable(*n* = 1657)	Increasing(*n* = 462)	Decreasing(*n* = 244)	High stable(*n* = 1603)	P
Glucose mg/dL (mean (SD))	100.59(15.14)	99.06(12.26)	107.94(16.71)	106.52(16.83)	< 0.001
Cholesterol mg/dL (mean (SD))	186.79(35.08)	190.69(36.58)	197.54(39.72)	196.81(36.59)	< 0.001
TG mg/dL (mean (SD))	89.69(42.27)	90.50(36.43)	146.94(65.73)	149.55(69.88)	< 0.001
HDL mg/dL (mean (SD))	58.27(15.62)	54.87(12.63)	49.52(12.91)	46.18(12.15)	< 0.001
LDL mg/dL (mean (SD))	112.32(30.79)	119.47(32.32)	119.19(35.48)	120.68(34.14)	< 0.001
hsCRP mg/L (mean (SD))	1.29(1.56)	1.31(1.45)	1.38(1.51)	1.62(1.46)	< 0.001
Age (mean (SD))	60.03(8.81)	57.82(8.60)	58.25(8.21)	56.61(7.90)	< 0.001
BMI kg/m2 (mean (SD))	20.44(1.81)	22.37(1.51)	23.82(1.74)	26.29(2.51)	< 0.001
SBP mmHg (mean (SD))	124.94(20.97)	126.34(24.36)	129.49(27.09)	132.67(24.98)	< 0.001
DBP mmHg (mean (SD))	72.09(11.16)	74.25(10.95)	74.88(11.84)	78.03(11.93)	< 0.001
TyG_index (mean (SD))	8.31(0.44)	8.33(0.41)	8.87(0.48)	8.87(0.50)	< 0.001
TyG_BMI (mean (SD))	169.79(16.21)	185.91(10.54)	210.76(12.28)	233.07(25.17)	< 0.001
Gender = Female (%)	741(44.7)	267(57.8)	131(53.7)	1013(63.2)	< 0.001
Residence (%)					< 0.001
Agricultural	1509(91.1)	410(88.7)	204(83.6)	1345(83.9)	
Non-Agricultural	143(8.6)	49(10.6)	39(16.0)	246(15.3)	
Unified Residence Hukou	5(0.3)	3(0.6)	1(0.4)	12(0.7)	
Marital status (%)					0.003
Married with spouse present	1397(84.3)	393(85.1)	206(84.4)	1430(89.2)	
Married but not living with spouse temporarily for reasons such as work	62(3.7)	18(3.9)	9(3.7)	55(3.4)	
Separated	8(0.5)	3(0.6)	2(0.8)	4(0.2)	
Divorced	14(0.8)	0(0.0)	0(0.0)	5(0.3)	
Widowed	163(9.8)	45(9.7)	26(10.7)	107(6.7)	
Never married	13(0.8)	3(0.6)	1(0.4)	2(0.1)	
Education (%)					< 0.001
No formal education illiterate	485(29.3)	141(30.5)	66(27.0)	446(27.8)	
Did not finish primary school but capable of reading or writing	362(21.8)	75(16.2)	41(16.8)	265(16.5)	
Sishu	11(0.7)	3(0.6)	0(0.0)	3(0.2)	
Elementary school	375(22.6)	112(24.2)	54(22.1)	352(22.0)	
Middle school	305(18.4)	94(20.3)	52(21.3)	367(22.9)	
High school	91(5.5)	28(6.1)	16(6.6)	127(7.9)	
Vocational school	15(0.9)	3(0.6)	8(3.3)	26(1.6)	
Two/Three Year College / Associate degree	11(0.7)	4(0.9)	6(2.5)	17(1.1)	
Four Year College / Bachelors degree	2(0.1)	2(0.4)	1(0.4)	0(0.0)	
Hypertension, n (%)	168(10.1)	62(13.4)	36(14.8)	446(27.8)	< 0.001
Dyslipidemia, n (%)	46(2.8)	22(4.8)	19(7.8)	163(10.2)	< 0.001
Diabetes, n (%)	25(1.5)	8(1.7)	10(4.1)	68(4.2)	< 0.001
Kidney, n (%)	108(6.5)	25(5.4)	16(6.6)	73(4.6)	0.093
Liver, n (%)	57(3.4)	13(2.8)	11(4.5)	54(3.4)	0.704
Smoking status (%)					< 0.001
Smokers	674(40.7)	138(29.9)	69(28.3)	345(21.5)	
Non-smokers	870(52.5)	298(64.5)	160(65.6)	1138(71.0)	
Ex-smokers	113(6.8)	26(5.6)	15(6.1)	120(7.5)	
Drinking status (%)					< 0.001
Drink more than once a month	514(31.0)	99(21.4)	69(28.3)	338(21.1)	
Drink but less than once a month	147(8.9)	34(7.4)	15(6.1)	142(8.9)	
None of these	996(60.1)	329(71.2)	160(65.6)	1123(70.1)	

Abbreviation: TG, Triglycerides; TyG, Triglyceride glucose; SBP, Systolic blood pressure; DBP, Diastolic blood pressure; HDL, High-density lipoprotein; LDL, low-density lipoprotein; hsCRP, Immunoturbidimetric assay; BMI, Body mass index.

### Association between TyG trajectory and risk of CVD

**Fig. 3:**
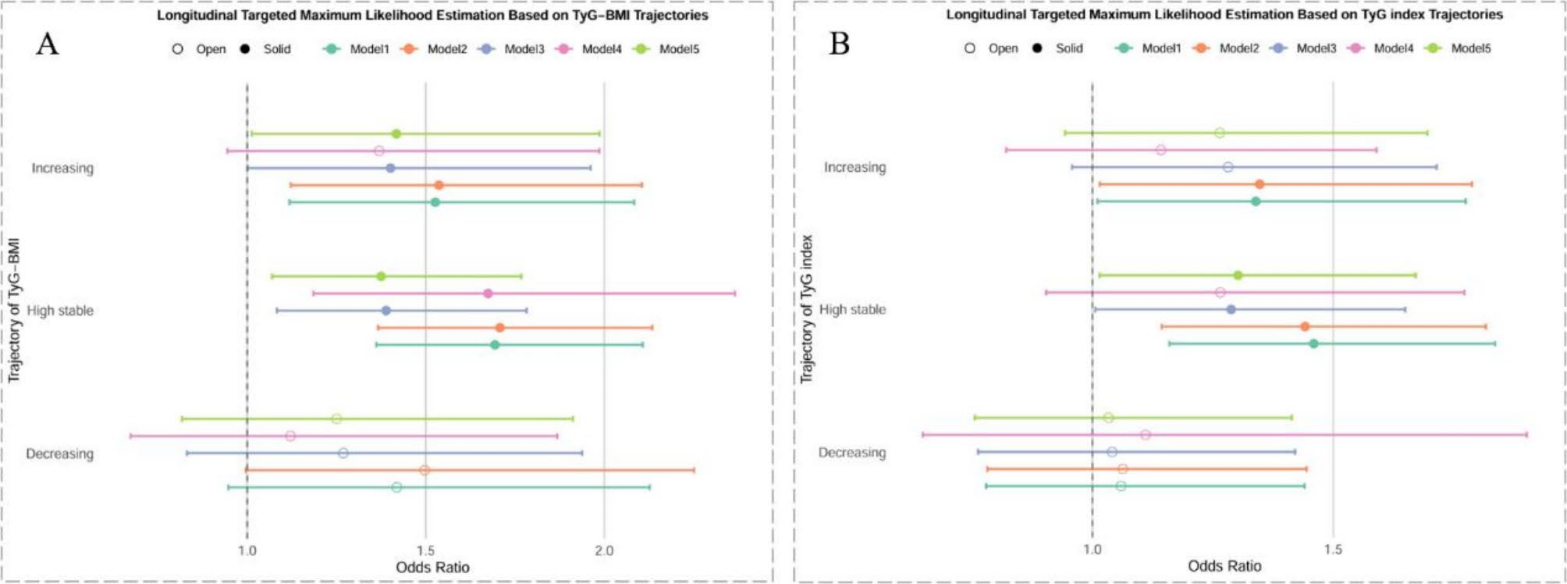
Longitudinal Targeted Maximum Likelihood Estimation based on TyG-BMI and TyG index trajectories. (1) **A** shows the estimated OR for TyG-BMI trajectories; **B** shows the estimated OR for TyG index.(2) An open dot indicates the ‘p-value’ is more than 0.05, while a solid dot indicates the significant result with a ‘p-value’ less than 0.05.(3) Model 1 was adjusted for age and gender; Model 2 further adjusted for model 1 covariates plus residence, baseline marital status, education, smoke status and drinking status; Model 3 further adjusted for Model 2 covariates plus comorbidities, including hypertension, dyslipidemia, diabetes, kidney; Model 4 further adjusted for Model 2 covariates plus laboratory parameters, including high-density lipoprotein, low-density lipoprotein, and high-sensitivity C-reactive protein (hsCRP). Model 5 further adjusted for the covariates based on Model 3, in addition to liver comorbidity, with marital status treated as a time-varying covariate and smoking status categorized as ex-smoker, current smoker, and non-smoker

Figure 3 presents the findings from the longitudinal targeted maximum likelihood estimation (LTMLE) analysis based on Model 1 with SuperLearner Library 1. The results demonstrated that participants in the ‘high stable’ and ‘increasing’ trajectory groups had a significantly higher risk of developing CVD compared to those in the ‘low stable’ group. Conversely, no significant differences were observed for the ‘decreasing’ group relative to the ‘low stable’ group.

When the exposure variable was TyG-BMI, individuals in the ‘high stable’ group exhibited 1.694 times higher odds of developing CVD (95% CI: 1.361–2.108) compared to the ‘low stable’ group in Model 1. Similar associations were identified in other models, with odds ratios (ORs) of 1.708 (95% CI: 1.367–2.134) in Model 2, 1.389 (95% CI: 1.083–1.782) in Model 3, 1.675 (95% CI: 1.185–2.366) in Model 4, and 1.375 (95% CI: 1.07–1.768) in Model 5. For the ‘increasing’ group, the odds of CVD were also elevated, albeit to a lesser extent. The ORs were 1.527 (95% CI: 1.119–2.084) in Model 1, 1.537 (95% CI: 1.122–2.106) in Model 2, 1.402 (95% CI: 1.001–1.962) in Model 3, 1.370 (95% CI: 0.945–1.986) in Model 4, and 1.418 (95% CI:1.012–1.986) in Model 5.

When the exposure was TyG index, the pattern remained consistent, although the magnitude of the odds ratios was slightly lower. For the ‘high stable’ group, the ORs were 1.459 (95% CI: 1.160–1.836) in Model 1, 1.441 (95% CI: 1.144–1.817) in Model 2, 1.288 (95% CI: 1.006–1.649) in Model 3, 1.266 (95% CI: 0.904–1.772) in Model 4, and 1.302 (95% CI: 1.015–1.671) in Model 5. Similarly, the ‘increasing’ group showed elevated odds of CVD compared to the ‘low stable’ group, with ORs of 1.339 (95% CI: 1.011–1.775) in Model 1, 1.347 (95% CI: 1.015–1.788) in Model 2, 1.282 (95% CI: 1.001–1.962) in Model 3, 1.142 (95% CI: 0.821–1.590) in Model 4, and 1.265 (95% CI: 0.943–1.696) in Model 5.

In summary, participants in the ‘high stable’ and ‘increasing’ groups consistently showed a significantly higher risk of CVD compared to the ‘low stable’ group, regardless of whether the exposure was TyG-BMI or TyG index. In contrast, the risk for the ‘decreasing’ group did not differ significantly from the ‘low stable’ group. Results from sensitivity analyses using alternative SuperLearner libraries are provided in Fig. S1–S6.

### Sensitivity analysis

The G-computation method was employed as an additional sensitivity analysis, yielding results that were largely consistent with those obtained from the LTMLE approach (Table 4). For TyG-BMI trajectories, participants in the ‘high stable’ and ‘increasing’ groups exhibited a significantly elevated risk of CVD compared to those in the ‘low stable’ group. In contrast, no significant differences were detected between the ‘decreasing’ and ‘low stable’ groups. Specifically, the G-computation method estimated that the odds of CVD in the ‘high stable’ group were 1.686 times higher than in the ‘low stable’ group (95% CI: 1.361–2.089), aligning with the findings from the LTMLE analysis. Similarly, for the ‘increasing’ group versus the ‘low stable’ group, Model 1 of the G-computation method estimated an odds ratio of 1.389 (95% CI: 1.007–1.915), slightly lower than the corresponding LTMLE results but still demonstrating consistency. Detailed results across various models adjusting for additional time-varying and time-invariant confounders are provided in Table 4.

**Table 4:** Comparison of the effects of TyG-BMI trajectories on CVD risk using ltmle and G-computation methods with superlearner library 1

Models	Trajectories	Methods	Estimate_OR	Std.dev_OR	Pvalue_OR	CI_OR_lower	CI_OR_upper	E_value
Model1	Decreasing	LTMLE	1.419	0.207	9.02E-02	0.947	2.127	1.000
		G-computation	1.214	0.224	3.85E-01	0.784	1.882	1.000
	High stable	LTMLE	1.694	0.112	***2.32E-06***	1.361	2.108	2.778
		G-computation	1.686	0.109	***1.78E-06***	1.361	2.089	2.761
	Increasing	LTMLE	1.527	0.159	***7.66E-03***	1.119	2.084	2.424
		G-computation	1.389	0.164	***4.52E-02***	1.007	1.915	2.124
Model2	Decreasing	LTMLE	1.497	0.208	5.27E-02	0.995	2.251	1.000
		G-computation	1.217	0.232	3.97E-01	0.773	1.917	1.000
	High stable	LTMLE	1.708	0.114	***2.51E-06***	1.367	2.134	2.808
		G-computation	1.684	0.111	***2.82E-06***	1.354	2.094	2.757
	Increasing	LTMLE	1.537	0.161	***7.46E-03***	1.122	2.106	2.445
		G-computation	1.384	0.166	***4.98E-02***	1.000	1.915	2.113
Model3	Decreasing	LTMLE	1.269	0.216	2.70E-01	0.831	1.938	1.000
		G-computation	1.064	0.234	7.91E-01	0.672	1.684	1.000
	High stable	LTMLE	1.389	0.127	***9.75E-03***	1.083	1.782	2.124
		G-computation	1.389	0.123	***7.71E-03***	1.091	1.768	2.124
	Increasing	LTMLE	1.402	0.172	***4.91E-02***	1.001	1.962	2.153
		G-computation	1.305	0.175	1.29E-01	0.926	1.841	1.000
Model4	Decreasing	LTMLE	1.121	0.260	6.60E-01	0.673	1.868	1.000
		G-computation	1.182	0.235	4.77E-01	0.746	1.873	1.000
	High stable	LTMLE	1.675	0.176	***3.47E-03***	1.185	2.366	2.738
		G-computation	1.587	0.180	***1.02E-02***	1.116	2.258	2.552
	Increasing	LTMLE	1.370	0.190	9.71E-02	0.945	1.986	1.000
		G-computation	1.344	0.179	9.87E-02	0.946	1.908	1.000
Model5	Decreasing	LTMLE	1.251	0.217	3.02E-01	0.818	1.913	1.000
		G-computation	1.049	0.235	8.40E-01	0.661	1.662	1.000
	High stable	LTMLE	1.375	0.128	***1.29E-02***	1.07	1.768	2.093
		G-computation	1.37	0.124	***1.08E-02***	1.075	1.745	2.082
	Increasing	LTMLE	1.418	0.172	***4.23E-02***	1.012	1.986	2.188
		G-computation	1.307	0.174	1.25E-01	0.928	1.839	1.000

1)SuperLearner library 1 includes generalized linear model in both outcome and exposure modeling in LTMLE

2)Abbreviation: OR, Odds Ratio; CI, Confidence interval

3)Model 1 was adjusted for age and gender; Model 2 further adjusted for model 1 covariates plus residence, baseline marital status, education, smoke status and drinking status; Model 3 further adjusted for Model 2 covariates plus comorbidities, including hypertension, dyslipidemia, diabetes, kidney; Model 4 further adjusted for Model 2 covariates plus laboratory parameters, including high-density lipoprotein, low-density lipoprotein, and high-sensitivity C-reactive protein (hsCRP). Model 5 further adjusted for the covariates based on Model 3, in addition to liver comorbidity, with marital status treated as a time-varying covariate and smoking status categorized as ex-smoker, current smoker, and non-smoker

4）Statistical significance was defined as a p-value less than 0.05, which is indicated in bold.

Furthermore, G-computation was applied to analyze TyG index trajectories as well, accounting for time-varying and time-invariant confounders across multiple models and utilizing alternative SuperLearner libraries. Changes in the SuperLearner configurations did not substantially affect the outcomes, underscoring the robustness of the findings. Comprehensive results are presented in Tables S3–S9.

In addition to the primary IR surrogates used in this study (TyG index and TyG-BMI), we also examined four additional IR markers—MetS-IR, SPISE, TG/HDL-C, and TyG-WHtR—to assess their impact on CVD risk. Generally, the results from these markers were consistent with those derived from the TyG index and TyG-BMI. In Model 1, when MetS-IR was used as the exposure, the odds ratio (OR) for comparing the ‘high stable’ group to the ‘low stable’ group was 1.473 (95% CI: 1.189–1.825). This increased slightly to 1.477 (95% CI: 1.180–1.848) when TG/HDL-C was used as the exposure. As anticipated, when TyG-WHtR was the exposure, the OR was 1.735 (95% CI: 1.377–2.186). In contrast, SPISE showed a protective effect, with an OR of 0.582 (95% CI: 0.467–0.725). A summary of the characteristics of these variables is provided in Table S10, while detailed results are presented in Table S11.

## Discussion

In this study, we employed a longitudinal TMLE framework to elucidate the relationship between dynamic trajectories of IR, as measured by the TyG index and the TyGBMI, and the incidence of CVD in a nationwide Chinese cohort. To best of our knowledge, this study is the first to explore the association between long-term TyG trajectories and CVD risk in a large Chinese national cohort, adjusting for time-varying confounders. Our findings underscore the significance of monitoring longitudinal changes in IR markers, which could enhance risk stratification and inform intervention strategies.

### Main findings

Our analysis indicated that individuals with stable high or increasing trajectories of TyG-BMI (and TyG index) face a significantly higher risk of developing CVD compared to those with stable low trajectories. This finding aligns with existing research [36] suggesting that persistent insulin resistance is a key factor in driving atherosclerosis and cardiovascular morbidity. Specifically, an increasing trajectory of the TyG index was linked to a heightened odds ratio for CVD, reinforcing the idea that fluctuations in insulin resistance over time are critical indicators of cardiovascular risk [14].

These results underscore the necessity of viewing insulin resistance as a dynamic rather than static construct. Traditional methodologies often depend on single-point measurements, which may overlook the temporal variations that accurately reflect changes in metabolic health [36, 37]. Our study design recognizes that insulin resistance can vary due to multiple factors, including lifestyle changes and disease progression. By adopting a longitudinal approach, we provide a more nuanced understanding of how these trajectories relate to cardiovascular outcomes.

While previous studies [38] have identified links between insulin resistance and adverse cardiovascular outcomes, many have been limited by cross-sectional data or single-time-point assessments. Our findings contribute to this body of work by demonstrating that the trajectory of insulin resistance—particularly when assessed through the TyG-BMI and TyG index—offers a strong predictive capability for CVD outcomes. This is consistent with recent research [39] emphasizing the importance of continuous monitoring of metabolic markers in high-risk populations, especially since insulin resistance is influenced by aging, obesity, and lifestyle factors. The components of the TyG index, including glucose and triglycerides, typically increase with age due to alterations in body fat, insulin signaling, and lipid metabolism [40]; Therefore, it is important to monitor the TyG index and TyG-BMI over time. Moreover, fluctuations in glucose levels and elevated plasma triglycerides are linked to an increased risk of CVD, emphasizing the need to account for these temporal changes [41, 42].

Recent studies [43] have shown the significance of tracking changes in the TyG index over time. For instance, in individuals with type 2 diabetes mellitus, both the baseline TyG index and its trajectories have been associated with major adverse cardiovascular events. Similarly, Xu et al. [44] identified three distinct TyG trajectories (low, moderate, and high) in a younger population, providing valuable insights into cardiovascular risk. Building on these findings, our study employed a more advanced causal method, adjusting for both time-invariant and time-varying confounders. By analyzing the dynamic changes in the TyG index and TyG-BMI, we gained a more comprehensive understanding of how these fluctuations impact long-term cardiovascular outcomes, thereby guiding and improving intervention strategies.

Utilizing the LTMLE framework allowed us to address complex confounding factors that could bias causal estimates in longitudinal studies. The doubly robust nature of LTMLE strengthens the validity of our findings, as it provides consistent estimates even if one of the models (either the outcome model or the exposure mechanism) is misspecified. This methodological rigor is particularly vital in our study, where time-varying confounders, such as other cardiometabolic biomarkers and lifestyle changes, could significantly impact the observed relationships.

### Clinical implications

Our findings have several clinical implications. We observed that both individuals with stable high or increasing trajectories of TyG-BMI (and TyG index) face a significantly higher risk of developing CVD compared to those with stable low trajectories. By recognizing dynamic insulin resistance (IR) trajectories as key indicators of CVD risk, healthcare professionals can more effectively customize preventive strategies and interventions for individuals with worsening metabolic profiles. Moreover, incorporating the TyG index and TyG-BMI into routine clinical assessments may enable the early identification of patients at increased risk for cardiovascular events.

On the other hand, the Odds of CVD from LTMLE is slightly larger than the results from G-computation, showing higher statistically power. It’s reasonable that LTMLE are double robust, and showing more reliable results. All the significant E-value is more than 2, indicating that the association is moderately robust to unmeasured confounding. It suggests that for the observed association to be fully explained by unmeasured confounding, the confounder would need to be relatively strong. However, causal conclusion should be drawn carefully and the further RCT is needed to verify if TyG-BMI and TyG index changes are causal risk factors of CVD.

Furthermore, when comparing TyG-BMI and TyG index as exposures, TyG-BMI consistently exhibited higher odds ratios (ORs) across nearly all scenarios in our study (Fig. S7-S10). This finding suggests that by incorporating BMI, TyG-BMI offers a more holistic representation of metabolic health, reflecting the combined effects of adiposity and insulin resistance (IR). This integration may explain the stronger associations (higher ORs) observed between TyG-BMI trajectories and CVD risk compared to the TyG index, which primarily captures IR. Additionally, TyG-BMI may more effectively reflect temporal changes in metabolic health, as BMI typically increases with age, intensifying IR and escalating cardiovascular risk over time. As a result, TyG-BMI trajectories might serve as a more sensitive indicator of cumulative metabolic burden and its influence on cardiovascular outcomes [45–47].

The mechanisms connecting changes in the TyG index and TyG-BMI are not yet fully understood, although several potential explanations have been suggested. Elevated TyG index values indicate worsening insulin resistance, which leads to an increased circulating level of glucose and triglycerides. This dyslipidemia contributes to the formation of atherosclerotic plaques by promoting endothelial dysfunction and vascular inflammation. Similarly, increased TyG-BMI values reflect the combination of insulin resistance and obesity, both of which contribute to adiposity-driven inflammatory responses that accelerate atherosclerosis [48, 49]. Future research should aim to uncover the underlying mechanisms linking IR trajectories to CVD outcomes. Investigating how factors such as inflammation, oxidative stress, and adiposity interact with insulin resistance may provide deeper insights into the pathophysiological processes contributing to CVD. Furthermore, longitudinal studies that include diverse populations and various lifestyle interventions would help enhance the broader applicability of our findings.

### Strengths and limitations

Our study has several notable strengths. First, our study captures ***dynamic changes in insulin resistance over time***, addressing limitations associated with cross-sectional studies that rely solely on single-point measurements. This design facilitates a more accurate assessment of how variations in insulin resistance impact cardiovascular risk. Second, the application of LTMLE provides a ***doubly robust framework*** for estimating causal effects, meaning that it is consistent ***if either the outcome model or the treatment model is correctly specified***. This approach effectively ***accounts for time-varying confounders***, offering more reliable estimates than traditional methods such as Cox regression or generalized estimating equations. Third, by examining ***both the TyG index and TyG-BMI as surrogate markers for insulin resistance***, the study provides a more nuanced understanding of cardiovascular health. To our knowledge, very few nationwide longitudinal studies accounted for exposure sequences while adjusting for time-varying confounders, making our study ***a valuable template for future research in this under-explored area***.

However, our study has several limitations. Although the CHARLS cohort is representative of the older Chinese population, the findings may not be directly applicable to other populations or ethnic groups. Differences in lifestyle, dietary patterns, and healthcare accessibility could restrict the generalizability of our results beyond China. *Secondly*, since medical records were not available in the CHARLS, the diagnosis of cardiovascular events in our study relied on self-reporting, which constitutes a limitation. In some other large-scale studies, such as the ELSA, have demonstrated that self-reported incidents of cardiovascular disease exhibit good agreement with medical records [50]. Evidence derived from clinical records represents a promising avenue for future research. *Thirdly*, distinguishing insulin resistance in individuals with and without diabetes is crucial, as the latter may compensate with increased insulin secretion, which is not possible in those with diabetes. Our study’s small sample size of only 111 participants with diabetes limited our ability to assess the differential impact of IR trajectories on CVD risk. Future research with larger samples is needed to explore this important issue further. *Additionally*, while the TyG index, TyG-BMI, and other markers (like MetS-IR, SPISE etc.) serve as valuable proxies for insulin resistance, they may not fully capture the complexity of metabolic dysregulation. Relying solely on these measures could omit other critical factors contributing to cardiovascular risk. *Furthermore*, as an observational study, our findings are susceptible to residual confounding despite the application of advanced statistical methods. Causal relationships cannot be firmly established due to potential biases inherent in observational designs. Nevertheless, we employed the E-value method to assess the robustness of our results. However, further randomized controlled trials (RCTs) are necessary to establish causal relationships conclusively, and the underlying mechanisms will warrant further studies.

## Conclusion

In conclusion, our study underscores the critical role of longitudinal trajectories of insulin resistance in predicting cardiovascular disease. By employing a rigorous analytical framework, we provide compelling evidence for the need to monitor IR dynamically, facilitating improved risk stratification and intervention strategies. As the burden of cardiovascular disease continues to rise, particularly in populations at risk, our findings highlight the imperative to advance research methodologies and clinical practices that reflect the complexities of cardiovascular health.

## Electronic supplementary material

Below is the link to the electronic supplementary material.

Supplementary Material 1

Supplementary Material 2

## Data Availability

The datasets used in this investigation are available in online repositories. Detailed descriptions of each survey and corresponding data have been published at http://charls.pku.edu.cn/.

## Abbreviations

CVD: Cardiovascular disease
IR: Insulin resistance
HOMA-IR: Homeostatic model assessment for insulin resistance
TyG: Triglyceride-glucose
MetS-IR: Metabolic score for insulin resistance
SPISE: Single-point insulin sensitivity estimator
TG/HDL-C: Triglycerides to HDL-C ratio
TyG-WHtR: TyG waist circumference-to-height ratio
CHARLS: China Health and Retirement Longitudinal Study
ICD-10: International Classification of Diseases, tenth revision
BMI: Body mass index
SBP: Systolic blood pressure
DBP: Diastolic blood pressure
HDL: High-density lipoprotein
LDL: Low-density lipoprotein
hsCRP: High-sensitivity C-reactive protein
DM: Diabetes mellitus
HTN: Hypertension
DLD: Dyslipidemia
OR: Odds ratio
CI: Confidence interval
TMLE: Targeted maximum likelihood estimation
LTMLE: Longitudinal targeted maximum likelihood estimation
