# Supplementary material for: Assessing the impact of insulin resistance trajectories on cardiovascular disease risk using longitudinal targeted maximum likelihood estimation
Source: Cardiovasc Diabetol. 2025 Mar 10;24:112. doi: 10.1186/s12933-025-02651-6 (PMC11895167; doi:10.1186/s12933-025-02651-6)
Supplement: Supplementary file 1 — Supplementary Material 1 [file 12933_2025_2651_MOESM1_ESM.docx]

**Supplementary Files (Tables and Figures)**

All supplementary files are available at:

<https://drive.google.com/drive/folders/1XGo7OOgpYRbBDnXfjOC5sQHAwi_hxn57?usp=drive_link>

**Supplementary Tables**

| Table S1 Definition of TyG index and TyG-BMI trajectories in our study | | | |
| --- | --- | --- | --- |
| Exposure | The first time point | The second time point | Trajectories |
| TyG index | 0 | 0 | Low stable |
|  | 0 | 1 | Increasing |
|  | 1 | 0 | Decreasing |
|  | 1 | 1 | High stable |
| TyG-BMI | 0 | 0 | Low stable |
|  | 0 | 1 | Increasing |
|  | 1 | 0 | Decreasing |
|  | 1 | 1 | High stable |
| 1) Abbreviation: TyG, Triglyceride glucose; BMI, Body mass index.  2) The median was used to dichotomize continuous exposure variables. Values above or equal to the median were coded as 1, while those below the median were coded as 0. | | | |

Table S2 Missing rate of the covariate in our study

| Factors | Missing Rate |
| --- | --- |
| Glucose | 0.00000 |
| Cholesterol | 0.00000 |
| TG | 0.00000 |
| HDL | 0.00000 |
| LDL | 0.00014 |
| hsCRP | 0.00000 |
| Gender_1M_2F | 0.00000 |
| Age | 0.00000 |
| Residence | 0.00000 |
| Marry | 0.00000 |
| Education | 0.00000 |
| BMI | 0.00000 |
| SBP | 0.00907 |
| DBP | 0.01088 |
| Hypertension | 0.00377 |
| Dyslipidemia | 0.01884 |
| Diabetes | 0.00768 |
| Kidney | 0.00251 |
| Smoke | 0.00014 |
| Drink | 0.00000 |
| TyG index | 0.00000 |
| TyG-BMI | 0.00000 |

Abbreviations: TG, Triglycerides; TyG, Triglyceride glucose; SBP, Systolic blood pressure; DBP, Diastolic blood pressure; HDL, High-density lipoprotein; LDL, low-density lipoprotein; hsCRP, Immunoturbidimetric assay; BMI, Body mass index.

| Table S3 Comparison of the effects of TyG-BMI trajectories on CVD risk using ltmle and G-computation methods with superlearner library 2 | | | | | | | | |
| --- | --- | --- | --- | --- | --- | --- | --- | --- |
| Models | Trajectories | Methods | Estimate_OR | Std.dev_OR | Pvalue_OR | CI_OR_lower | CI_OR_upper | E_value |
| Model1 | Decreasing | LTMLE | 1.417 | 0.205 | 8.93E-02 | 0.948 | 2.117 | 1 |
|  |  | G-computation | 1.175 | 0.218 | 4.61E-01 | 0.766 | 1.801 | 1 |
|  | High stable | LTMLE | 1.692 | 0.112 | ***2.55E-06*** | 1.359 | 2.106 | 2.774 |
|  |  | G-computation | 1.595 | 0.108 | ***1.58E-05*** | 1.29 | 1.972 | 2.569 |
|  | Increasing | LTMLE | 1.529 | 0.159 | ***7.47E-03*** | 1.12 | 2.087 | 2.428 |
|  |  | G-computation | 1.349 | 0.163 | 6.68E-02 | 0.979 | 1.857 | 1 |
| Model2 | Decreasing | LTMLE | 1.492 | 0.205 | 5.12E-02 | 0.998 | 2.231 | 1 |
|  |  | G-computation | 1.201 | 0.21 | 3.84E-01 | 0.796 | 1.813 | 1 |
|  | High stable | LTMLE | 1.71 | 0.114 | ***2.36E-06*** | 1.368 | 2.136 | 2.812 |
|  |  | G-computation | 1.537 | 0.108 | ***6.55E-05*** | 1.245 | 1.898 | 2.445 |
|  | Increasing | LTMLE | 1.536 | 0.16 | ***7.30E-03*** | 1.123 | 2.102 | 2.443 |
|  |  | G-computation | 1.337 | 0.156 | 6.18E-02 | 0.986 | 1.814 | 1 |
| Model3 | Decreasing | LTMLE | 1.308 | 0.212 | 2.05E-01 | 0.863 | 1.981 | 1 |
|  |  | G-computation | 1.112 | 0.202 | 6.01E-01 | 0.748 | 1.653 | 1 |
|  | High stable | LTMLE | 1.397 | 0.125 | ***7.40E-03*** | 1.094 | 1.784 | 2.142 |
|  |  | G-computation | 1.275 | 0.114 | ***3.38E-02*** | 1.019 | 1.595 | 1.867 |
|  | Increasing | LTMLE | 1.421 | 0.169 | ***3.71E-02*** | 1.021 | 1.978 | 2.194 |
|  |  | G-computation | 1.248 | 0.157 | 1.60E-01 | 0.916 | 1.699 | 1 |
| Model4 | Decreasing | LTMLE | 1.324 | 0.252 | 2.67E-01 | 0.807 | 2.171 | 1 |
|  |  | G-computation | 1.156 | 0.064 | ***2.44E-02*** | 1.019 | 1.311 | 1.581 |
|  | High stable | LTMLE | 1.548 | 0.16 | ***6.22E-03*** | 1.132 | 2.116 | 2.469 |
|  |  | G-computation | 1.446 | 0.067 | ***2.97E-08*** | 1.269 | 1.648 | 2.249 |
|  | Increasing | LTMLE | 1.433 | 0.197 | 6.78E-02 | 0.974 | 2.107 | 1 |
|  |  | G-computation | 1.284 | 0.067 | ***1.82E-04*** | 1.126 | 1.463 | 1.888 |
| Model5 | Decreasing | LTMLE | 1.255 | 0.212 | 2.85E-01 | 0.828 | 1.902 | 1.000 |
|  |  | G-computation | 1.088 | 0.179 | 6.36E-01 | 0.766 | 1.546 | 1.000 |
|  | High stable | LTMLE | 1.386 | 0.126 | ***9.35E-03*** | 1.084 | 1.773 | 2.117 |
|  |  | G-computation | 1.245 | 0.108 | ***4.35E-02*** | 1.006 | 1.539 | 1.797 |
|  | Increasing | LTMLE | 1.423 | 0.169 | ***3.74E-02*** | 1.021 | 1.983 | 2.199 |
|  |  | G-computation | 1.245 | 0.143 | 1.27E-01 | 0.94 | 1.648 | 1.000 |
| (1)SuperLearner library 2 includes generalized linear model, mean estimation, lasso and elastic-net regularized generalized linear models, random forest, and xgboost in both outcome and exposure modeling in LTMLE. | | | | | | | | |
| (2) Abbreviation: OR, Odds Ratio; CI, Confidence interval. | | | | |  |  |  |  |

| Table S4 Comparison of the effects of TyG-BMI trajectories on CVD risk using ltmle and g-computation methods with superlearner library 3 | | | | | | | | |
| --- | --- | --- | --- | --- | --- | --- | --- | --- |
| Models | Trajectories | Methods | Estimate_OR | Std.dev_OR | Pvalue_OR | CI_OR_lower | CI_OR_upper | E_value |
| Model1 | Decreasing | LTMLE | 1.418 | 0.207 | 9.08E-02 | 0.946 | 2.126 | 1 |
|  |  | G-computation | 1.214 | 0.223 | 3.83E-01 | 0.785 | 1.878 | 1 |
|  | High stable | LTMLE | 1.693 | 0.112 | ***2.42E-06*** | 1.36 | 2.106 | 2.776 |
|  |  | G-computation | 1.686 | 0.109 | ***1.69E-06*** | 1.361 | 2.088 | 2.761 |
|  | Increasing | LTMLE | 1.529 | 0.158 | ***7.31E-03*** | 1.121 | 2.086 | 2.428 |
|  |  | G-computation | 1.389 | 0.164 | ***4.49E-02*** | 1.008 | 1.914 | 2.124 |
| Model2 | Decreasing | LTMLE | 1.513 | 0.205 | ***4.36E-02*** | 1.012 | 2.263 | 2.394 |
|  |  | G-computation | 1.229 | 0.226 | 3.63E-01 | 0.788 | 1.915 | 1 |
|  | High stable | LTMLE | 1.713 | 0.114 | ***2.25E-06*** | 1.371 | 2.141 | 2.818 |
|  |  | G-computation | 1.686 | 0.109 | ***1.77E-06*** | 1.361 | 2.09 | 2.761 |
|  | Increasing | LTMLE | 1.534 | 0.16 | ***7.58E-03*** | 1.121 | 2.101 | 2.439 |
|  |  | G-computation | 1.396 | 0.162 | ***3.91E-02*** | 1.017 | 1.917 | 2.14 |
| Model3 | Decreasing | LTMLE | 1.289 | 0.216 | 2.40E-01 | 0.844 | 1.967 | 1 |
|  |  | G-computation | 1.084 | 0.221 | 7.15E-01 | 0.703 | 1.673 | 1 |
|  | High stable | LTMLE | 1.397 | 0.127 | ***8.71E-03*** | 1.088 | 1.794 | 2.142 |
|  |  | G-computation | 1.386 | 0.114 | ***4.27E-03*** | 1.108 | 1.735 | 2.117 |
|  | Increasing | LTMLE | 1.422 | 0.172 | ***4.06E-02*** | 1.015 | 1.993 | 2.197 |
|  |  | G-computation | 1.312 | 0.166 | 1.02E-01 | 0.948 | 1.818 | 1 |
| Model4 | Decreasing | LTMLE | 1.296 | 0.253 | 3.06E-01 | 0.789 | 2.128 | 1 |
|  |  | G-computation | 1.18 | 0.142 | 2.42E-01 | 0.894 | 1.557 | 1 |
|  | High stable | LTMLE | 1.548 | 0.152 | ***3.97E-03*** | 1.15 | 2.085 | 2.469 |
|  |  | G-computation | 1.578 | 0.093 | ***9.44E-07*** | 1.315 | 1.894 | 2.533 |
|  | Increasing | LTMLE | 1.453 | 0.194 | 5.40E-02 | 0.994 | 2.124 | 1 |
|  |  | G-computation | 1.343 | 0.116 | ***1.09E-02*** | 1.07 | 1.685 | 2.22 |
| Model5 | Decreasing | LTMLE | 1.279 | 0.217 | 2.58E-01 | 0.835 | 1.957 | 1.000 |
|  |  | G-computation | 1.078 | 0.219 | 7.32E-01 | 0.702 | 1.654 | 1.000 |
|  | High stable | LTMLE | 1.383 | 0.129 | ***1.22E-02*** | 1.073 | 1.782 | 2.111 |
|  |  | G-computation | 1.366 | 0.112 | ***5.37E-03*** | 1.097 | 1.702 | 2.073 |
|  | Increasing | LTMLE | 1.436 | 0.173 | ***3.61E-02*** | 1.024 | 2.013 | 2.227 |
|  |  | G-computation | 1.308 | 0.158 | 9.02E-02 | 0.959 | 1.783 | 1.000 |
| (1) SuperLearner library 3 includes generalized linear model and xgboost in both outcome and exposure modeling in LTMLE. | | | | | | | | |
| (2) Abbreviation: OR, Odds Ratio; CI, Confidence interval. | | | | |  |  |  |  |

| Table S5 Comparison of the effects of TyG-BMI trajectories on CVD risk using ltmle and g-computation methods with superlearner library 4 | | | | | | | | |
| --- | --- | --- | --- | --- | --- | --- | --- | --- |
| Models | Trajectories | Methods | Estimate_OR | Std.dev_OR | Pvalue_OR | CI_OR_lower | CI_OR_upper | E_value |
| Model1 | Decreasing | LTMLE | 1.417 | 0.206 | 9.08E-02 | 0.946 | 2.123 | 1 |
|  |  | G-computation | 1.176 | 0.215 | 4.52E-01 | 0.771 | 1.794 | 1 |
|  | High stable | LTMLE | 1.692 | 0.112 | ***2.49E-06*** | 1.359 | 2.105 | 2.774 |
|  |  | G-computation | 1.597 | 0.108 | ***1.49E-05*** | 1.292 | 1.975 | 2.573 |
|  | Increasing | LTMLE | 1.529 | 0.159 | ***7.40E-03*** | 1.121 | 2.086 | 2.428 |
|  |  | G-computation | 1.341 | 0.163 | 7.27E-02 | 0.973 | 1.846 | 1 |
| Model2 | Decreasing | LTMLE | 1.509 | 0.205 | ***4.44E-02*** | 1.01 | 2.254 | 2.385 |
|  |  | G-computation | 1.18 | 0.208 | 4.27E-01 | 0.785 | 1.775 | 1 |
|  | High stable | LTMLE | 1.708 | 0.114 | ***2.52E-06*** | 1.366 | 2.134 | 2.808 |
|  |  | G-computation | 1.484 | 0.111 | ***3.59E-04*** | 1.195 | 1.843 | 2.331 |
|  | Increasing | LTMLE | 1.534 | 0.161 | ***7.69E-03*** | 1.12 | 2.101 | 2.439 |
|  |  | G-computation | 1.312 | 0.161 | 9.07E-02 | 0.958 | 1.798 | 1 |
| Model3 | Decreasing | LTMLE | 1.3 | 0.211 | 2.15E-01 | 0.859 | 1.968 | 1 |
|  |  | G-computation | 1.125 | 0.2 | 5.57E-01 | 0.76 | 1.664 | 1 |
|  | High stable | LTMLE | 1.404 | 0.126 | ***6.89E-03*** | 1.098 | 1.796 | 2.157 |
|  |  | G-computation | 1.268 | 0.115 | ***3.84E-02*** | 1.013 | 1.587 | 1.851 |
|  | Increasing | LTMLE | 1.405 | 0.168 | ***4.34E-02*** | 1.01 | 1.955 | 2.159 |
|  |  | G-computation | 1.246 | 0.151 | 1.45E-01 | 0.927 | 1.676 | 1 |
| Model4 | Decreasing | LTMLE | 1.313 | 0.251 | 2.78E-01 | 0.803 | 2.149 | 1 |
|  |  | G-computation | 1.144 | 0.064 | ***3.58E-02*** | 1.009 | 1.298 | 1.55 |
|  | High stable | LTMLE | 1.536 | 0.16 | ***7.39E-03*** | 1.122 | 2.103 | 2.443 |
|  |  | G-computation | 1.346 | 0.065 | ***4.69E-06*** | 1.185 | 1.529 | 2.028 |
|  | Increasing | LTMLE | 1.418 | 0.197 | 7.62E-02 | 0.964 | 2.085 | 1 |
|  |  | G-computation | 1.236 | 0.063 | ***7.40E-04*** | 1.093 | 1.397 | 1.776 |
| Model5 | Decreasing | LTMLE | 1.251 | 0.212 | 2.91E-01 | 0.825 | 1.896 | 1.000 |
|  |  | G-computation | 1.112 | 0.183 | 5.62E-01 | 0.777 | 1.592 | 1.000 |
|  | High stable | LTMLE | 1.386 | 0.127 | ***1.02E-02*** | 1.08 | 1.777 | 2.117 |
|  |  | G-computation | 1.269 | 0.113 | ***3.45E-02*** | 1.018 | 1.583 | 1.853 |
|  | Increasing | LTMLE | 1.42 | 0.169 | ***3.81E-02*** | 1.02 | 1.979 | 2.192 |
|  |  | G-computation | 1.231 | 0.141 | 1.42E-01 | 0.933 | 1.624 | 1.000 |
| (1)SuperLearner library 4 includes generalized linear model, mean estimation, random forest, and xgboost in both outcome and exposure modeling in LTMLE. | | | | | | | | |
| (2) Abbreviation: OR, Odds Ratio; CI, Confidence interval. | | | | |  |  |  |  |

| Table S6 Comparison of the effects of TyG index trajectories on CVD risk using ltmle and g-computation methods with superlearner library 1 | | | | | | | | |
| --- | --- | --- | --- | --- | --- | --- | --- | --- |
| Models | Trajectories | Methods | Estimate_OR | Std.dev_OR | Pvalue_OR | CI_OR_lower | CI_OR_upper | E_value |
| Model1 | Decreasing | LTMLE | 1.059 | 0.157 | 7.13E-01 | 0.779 | 1.440 | 1.000 |
|  |  | G-computation | 1.054 | 0.160 | 7.42E-01 | 0.771 | 1.441 | 1.000 |
|  | High stable | LTMLE | 1.459 | 0.117 | ***1.26E-03*** | 1.160 | 1.836 | 2.277 |
|  |  | G-computation | 1.447 | 0.119 | ***1.96E-03*** | 1.145 | 1.828 | 2.251 |
|  | Increasing | LTMLE | 1.339 | 0.144 | ***4.20E-02*** | 1.011 | 1.775 | 2.013 |
|  |  | G-computation | 1.373 | 0.144 | ***2.79E-02*** | 1.035 | 1.822 | 2.089 |
| Model2 | Decreasing | LTMLE | 1.063 | 0.156 | 6.96E-01 | 0.782 | 1.445 | 1.000 |
|  |  | G-computation | 1.049 | 0.160 | 7.67E-01 | 0.766 | 1.436 | 1.000 |
|  | High stable | LTMLE | 1.441 | 0.118 | ***1.95E-03*** | 1.144 | 1.817 | 2.238 |
|  |  | G-computation | 1.430 | 0.120 | ***2.96E-03*** | 1.130 | 1.811 | 2.214 |
|  | Increasing | LTMLE | 1.347 | 0.144 | ***3.89E-02*** | 1.015 | 1.788 | 2.031 |
|  |  | G-computation | 1.364 | 0.146 | ***3.36E-02*** | 1.024 | 1.816 | 2.069 |
| Model3 | Decreasing | LTMLE | 1.041 | 0.159 | 8.01E-01 | 0.762 | 1.421 | 1.000 |
|  |  | G-computation | 0.976 | 0.165 | 8.84E-01 | 0.707 | 1.348 | 1.000 |
|  | High stable | LTMLE | 1.288 | 0.126 | ***4.48E-02*** | 1.006 | 1.649 | 1.897 |
|  |  | G-computation | 1.246 | 0.128 | 8.58E-02 | 0.970 | 1.601 | 1.000 |
|  | Increasing | LTMLE | 1.282 | 0.148 | 9.47E-02 | 0.958 | 1.714 | 1.000 |
|  |  | G-computation | 1.276 | 0.149 | 1.03E-01 | 0.952 | 1.709 | 1.000 |
| Model4 | Decreasing | LTMLE | 1.110 | 0.275 | 7.04E-01 | 0.648 | 1.902 | 1.000 |
|  |  | G-computation | 1.002 | 0.318 | 9.96E-01 | 0.537 | 1.869 | 1.000 |
|  | High stable | LTMLE | 1.266 | 0.172 | 1.70E-01 | 0.904 | 1.772 | 1.000 |
|  |  | G-computation | 1.279 | 0.184 | 1.81E-01 | 0.892 | 1.834 | 1.000 |
|  | Increasing | LTMLE | 1.142 | 0.169 | 4.30E-01 | 0.821 | 1.590 | 1.000 |
|  |  | G-computation | 1.277 | 0.173 | 1.58E-01 | 0.910 | 1.792 | 1.000 |
| Model5 | Decreasing | LTMLE | 1.034 | 0.160 | 8.36E-01 | 0.756 | 1.414 | 1.000 |
|  |  | G-computation | 0.982 | 0.166 | 9.12E-01 | 0.709 | 1.360 | 1.000 |
|  | High stable | LTMLE | 1.302 | 0.127 | ***3.78E-02*** | 1.015 | 1.671 | 1.929 |
|  |  | G-computation | 1.260 | 0.130 | 7.62E-02 | 0.976 | 1.626 | 1.000 |
|  | Increasing | LTMLE | 1.265 | 0.150 | 1.17E-01 | 0.943 | 1.696 | 1.000 |
|  |  | G-computation | 1.283 | 0.151 | 9.86E-02 | 0.955 | 1.724 | 1.000 |
| (1) SuperLearner library 1 includes generalized linear model. | | | | |  |  |  |  |
| (2) Abbreviation: OR, Odds Ratio; CI, Confidence interval. | | | | |  |  |  |  |

| Table S7 Comparison of the effects of TyG index trajectories on CVD risk using ltmle and g-computation methods with superlearner library 2 | | | | | | | | |
| --- | --- | --- | --- | --- | --- | --- | --- | --- |
| Models | Trajectories | Methods | Estimate_OR | Std.dev_OR | Pvalue_OR | CI_OR_lower | CI_OR_upper | E_value |
| Model1 | Decreasing | LTMLE | 1.049 | 0.156 | 7.60E-01 | 0.772 | 1.425 | 1 |
|  |  | G-computation | 1.046 | 0.154 | 7.71E-01 | 0.774 | 1.413 | 1 |
|  | High stable | LTMLE | 1.452 | 0.117 | ***1.37E-03*** | 1.155 | 1.824 | 2.262 |
|  |  | G-computation | 1.356 | 0.117 | ***9.46E-03*** | 1.077 | 1.708 | 2.051 |
|  | Increasing | LTMLE | 1.34 | 0.143 | ***4.07E-02*** | 1.012 | 1.773 | 2.015 |
|  |  | G-computation | 1.303 | 0.143 | 6.39E-02 | 0.985 | 1.724 | 1 |
| Model2 | Decreasing | LTMLE | 1.078 | 0.155 | 6.29E-01 | 0.795 | 1.462 | 1 |
|  |  | G-computation | 1.016 | 0.149 | 9.14E-01 | 0.759 | 1.36 | 1 |
|  | High stable | LTMLE | 1.439 | 0.117 | ***1.86E-03*** | 1.144 | 1.81 | 2.234 |
|  |  | G-computation | 1.303 | 0.116 | ***2.23E-02*** | 1.038 | 1.636 | 1.931 |
|  | Increasing | LTMLE | 1.352 | 0.143 | ***3.51E-02*** | 1.021 | 1.789 | 2.042 |
|  |  | G-computation | 1.316 | 0.138 | ***4.58E-02*** | 1.005 | 1.723 | 1.961 |
| Model3 | Decreasing | LTMLE | 1.037 | 0.154 | 8.15E-01 | 0.766 | 1.402 | 1 |
|  |  | G-computation | 1.039 | 0.135 | 7.74E-01 | 0.798 | 1.354 | 1 |
|  | High stable | LTMLE | 1.276 | 0.118 | ***3.93E-02*** | 1.012 | 1.609 | 1.869 |
|  |  | G-computation | 1.185 | 0.113 | 1.34E-01 | 0.949 | 1.479 | 1 |
|  | Increasing | LTMLE | 1.272 | 0.143 | 9.18E-02 | 0.962 | 1.681 | 1 |
|  |  | G-computation | 1.21 | 0.129 | 1.40E-01 | 0.939 | 1.56 | 1 |
| Model4 | Decreasing | LTMLE | 0.979 | 0.198 | 9.16E-01 | 0.664 | 1.443 | 1 |
|  |  | G-computation | 1.007 | 0.06 | 9.13E-01 | 0.896 | 1.131 | 1 |
|  | High stable | LTMLE | 1.296 | 0.173 | 1.34E-01 | 0.923 | 1.818 | 1 |
|  |  | G-computation | 1.226 | 0.065 | ***1.77E-03*** | 1.079 | 1.394 | 1.752 |
|  | Increasing | LTMLE | 1.265 | 0.182 | 1.98E-01 | 0.884 | 1.808 | 1 |
|  |  | G-computation | 1.223 | 0.065 | ***1.87E-03*** | 1.077 | 1.388 | 1.745 |
| Model5 | Decreasing | LTMLE | 1.027 | 0.153 | 8.63E-01 | 0.76 | 1.387 | 1.000 |
|  |  | G-computation | 1.029 | 0.132 | 8.26E-01 | 0.795 | 1.333 | 1.000 |
|  | High stable | LTMLE | 1.281 | 0.118 | ***3.59E-02*** | 1.016 | 1.613 | 1.881 |
|  |  | G-computation | 1.184 | 0.11 | 1.26E-01 | 0.954 | 1.47 | 1.000 |
|  | Increasing | LTMLE | 1.266 | 0.141 | 9.46E-02 | 0.96 | 1.67 | 1.000 |
|  |  | G-computation | 1.202 | 0.121 | 1.27E-01 | 0.949 | 1.522 | 1.000 |
| (1) SuperLearner library 2 includes generalized linear model, mean estimation, lasso and elastic-net regularized generalized linear models, random forest, and xgboost in both outcome and exposure modeling in LTMLE. | | | | | | | | |
| (2) Abbreviation: OR, Odds Ratio; CI, Confidence interval. | | | | |  |  |  |  |

| Table S8 Comparison of the effects of TyG index trajectories on CVD risk using ltmle and g-computation methods with superlearner library 3 | | | | | | | | |
| --- | --- | --- | --- | --- | --- | --- | --- | --- |
| Models | Trajectories | Methods | Estimate_OR | Std.dev_OR | Pvalue_OR | CI_OR_lower | CI_OR_upper | E_value |
| Model1 | Decreasing | LTMLE | 1.062 | 0.156 | 7.01E-01 | 0.782 | 1.44 | 1.000 |
|  |  | G-computation | 1.054 | 0.159 | 7.41E-01 | 0.772 | 1.44 | 1.000 |
|  | High stable | LTMLE | 1.456 | 0.117 | ***1.25E-03*** | 1.159 | 1.83 | 2.271 |
|  |  | G-computation | 1.448 | 0.119 | ***1.81E-03*** | 1.148 | 1.827 | 2.253 |
|  | Increasing | LTMLE | 1.336 | 0.144 | ***4.39E-02*** | 1.008 | 1.77 | 2.006 |
|  |  | G-computation | 1.371 | 0.143 | ***2.74E-02*** | 1.036 | 1.816 | 2.084 |
| Model2 | Decreasing | LTMLE | 1.078 | 0.156 | 6.27E-01 | 0.795 | 1.463 | 1.000 |
|  |  | G-computation | 1.05 | 0.155 | 7.54E-01 | 0.774 | 1.424 | 1.000 |
|  | High stable | LTMLE | 1.443 | 0.117 | ***1.70E-03*** | 1.148 | 1.815 | 2.243 |
|  |  | G-computation | 1.433 | 0.117 | ***2.14E-03*** | 1.139 | 1.803 | 2.221 |
|  | Increasing | LTMLE | 1.359 | 0.143 | ***3.25E-02*** | 1.026 | 1.8 | 2.057 |
|  |  | G-computation | 1.368 | 0.139 | ***2.46E-02*** | 1.041 | 1.798 | 2.078 |
| Model3 | Decreasing | LTMLE | 1.037 | 0.156 | 8.15E-01 | 0.764 | 1.408 | 1.000 |
|  |  | G-computation | 0.979 | 0.15 | 8.88E-01 | 0.73 | 1.313 | 1.000 |
|  | High stable | LTMLE | 1.275 | 0.12 | ***4.25E-02*** | 1.008 | 1.612 | 1.867 |
|  |  | G-computation | 1.243 | 0.115 | 5.80E-02 | 0.993 | 1.557 | 1.000 |
|  | Increasing | LTMLE | 1.281 | 0.144 | 8.57E-02 | 0.966 | 1.7 | 1.000 |
|  |  | G-computation | 1.276 | 0.134 | 6.84E-02 | 0.982 | 1.657 | 1.000 |
| Model4 | Decreasing | LTMLE | 0.945 | 0.192 | 7.69E-01 | 0.649 | 1.376 | 1.000 |
|  |  | G-computation | 0.998 | 0.125 | 9.86E-01 | 0.782 | 1.274 | 1.000 |
|  | High stable | LTMLE | 1.259 | 0.159 | 1.48E-01 | 0.921 | 1.721 | 1.000 |
|  |  | G-computation | 1.278 | 0.11 | ***2.60E-02*** | 1.03 | 1.586 | 1.874 |
|  | Increasing | LTMLE | 1.232 | 0.175 | 2.32E-01 | 0.875 | 1.735 | 1.000 |
|  |  | G-computation | 1.278 | 0.118 | ***3.82E-02*** | 1.013 | 1.612 | 1.874 |
| Model5 | Decreasing | LTMLE | 1.033 | 0.155 | 8.35E-01 | 0.762 | 1.401 | 1.000 |
|  |  | G-computation | 0.98 | 0.148 | 8.92E-01 | 0.733 | 1.311 | 1.165 |
|  | High stable | LTMLE | 1.28 | 0.12 | ***3.92E-02*** | 1.012 | 1.618 | 1.879 |
|  |  | G-computation | 1.25 | 0.116 | 5.51E-02 | 0.995 | 1.569 | 1.000 |
|  | Increasing | LTMLE | 1.271 | 0.144 | 9.60E-02 | 0.958 | 1.686 | 1.000 |
|  |  | G-computation | 1.283 | 0.132 | 5.96E-02 | 0.99 | 1.663 | 1.000 |
| (1) SuperLearner library 3 includes generalized linear model and xgboost in both outcome and exposure modeling in LTMLE. | | | | | | | | |
| (2) Abbreviation: OR, Odds Ratio; CI, Confidence interval. | | | |  |  |  |  |  |

| Table S9 Comparison of the effects of TyG index trajectories on CVD risk using ltmle and g-computation methods with superlearner library 4 | | | | | | | | |
| --- | --- | --- | --- | --- | --- | --- | --- | --- |
| Models | Trajectories | Methods | Estimate_OR | Std.dev_OR | Pvalue_OR | CI_OR_lower | CI_OR_upper | E_value |
| Model1 | Decreasing | LTMLE | 1.048 | 0.157 | 7.65E-01 | 0.771 | 1.424 | 1.000 |
|  |  | G-computation | 1.046 | 0.154 | 7.70E-01 | 0.773 | 1.416 | 1.000 |
|  | High stable | LTMLE | 1.448 | 0.117 | ***1.51E-03*** | 1.152 | 1.819 | 2.253 |
|  |  | G-computation | 1.378 | 0.118 | ***6.69E-03*** | 1.093 | 1.738 | 2.100 |
|  | Increasing | LTMLE | 1.339 | 0.143 | ***4.15E-02*** | 1.011 | 1.772 | 2.013 |
|  |  | G-computation | 1.321 | 0.142 | 5.03E-02 | 1 | 1.746 | 1.000 |
| Model2 | Decreasing | LTMLE | 1.078 | 0.155 | 6.28E-01 | 0.795 | 1.461 | 1.000 |
|  |  | G-computation | 1.055 | 0.146 | 7.12E-01 | 0.793 | 1.404 | 1.000 |
|  | High stable | LTMLE | 1.433 | 0.117 | ***2.16E-03*** | 1.139 | 1.802 | 2.221 |
|  |  | G-computation | 1.302 | 0.116 | ***2.28E-02*** | 1.037 | 1.635 | 1.929 |
|  | Increasing | LTMLE | 1.359 | 0.143 | ***3.16E-02*** | 1.027 | 1.797 | 2.057 |
|  |  | G-computation | 1.269 | 0.138 | 8.30E-02 | 0.969 | 1.662 | 1.000 |
| Model3 | Decreasing | LTMLE | 1.043 | 0.154 | 7.86E-01 | 0.771 | 1.409 | 1.000 |
|  |  | G-computation | 1.041 | 0.132 | 7.60E-01 | 0.803 | 1.349 | 1.000 |
|  | High stable | LTMLE | 1.279 | 0.119 | ***3.84E-02*** | 1.013 | 1.614 | 1.876 |
|  |  | G-computation | 1.169 | 0.114 | 1.70E-01 | 0.935 | 1.461 | 1.000 |
|  | Increasing | LTMLE | 1.274 | 0.142 | 8.86E-02 | 0.964 | 1.683 | 1.000 |
|  |  | G-computation | 1.212 | 0.128 | 1.34E-01 | 0.942 | 1.559 | 1.000 |
| Model4 | Decreasing | LTMLE | 0.97 | 0.199 | 8.80E-01 | 0.657 | 1.433 | 1.000 |
|  |  | G-computation | 1.004 | 0.061 | 9.43E-01 | 0.892 | 1.131 | 1.000 |
|  | High stable | LTMLE | 1.262 | 0.173 | 1.78E-01 | 0.9 | 1.77 | 1.000 |
|  |  | G-computation | 1.162 | 0.065 | ***2.03E-02*** | 1.024 | 1.32 | 1.596 |
|  | Increasing | LTMLE | 1.257 | 0.183 | 2.11E-01 | 0.878 | 1.8 | 1.000 |
|  |  | G-computation | 1.171 | 0.061 | ***9.79E-03*** | 1.039 | 1.321 | 1.618 |
| Model5 | Decreasing | LTMLE | 1.04 | 0.153 | 7.96E-01 | 0.771 | 1.403 | 1.000 |
|  |  | G-computation | 1.032 | 0.132 | 8.12E-01 | 0.797 | 1.337 | 1.000 |
|  | High stable | LTMLE | 1.281 | 0.119 | ***3.73E-02*** | 1.015 | 1.616 | 1.881 |
|  |  | G-computation | 1.174 | 0.111 | 1.50E-01 | 0.944 | 1.459 | 1.000 |
|  | Increasing | LTMLE | 1.264 | 0.142 | 9.79E-02 | 0.958 | 1.669 | 1.000 |
|  |  | G-computation | 1.187 | 0.121 | 1.57E-01 | 0.936 | 1.506 | 1.000 |
| (1) SuperLearner library 4 includes generalized linear model, mean estimation, random forest, and xgboost in both outcome and exposure modeling in LTMLE. | | | | | | | | |
| (2) Abbreviation: OR, Odds Ratio; CI, Confidence interval. | | | | |  |  |  |  |

Table S10 Characteristics of additional IR markers classified by CVD status at follow-up

| Characteristics | Time points | Total (n=3966) | Non-CVD (n=3467) | CVD (n=499) | P |  |
| --- | --- | --- | --- | --- | --- | --- |
|  |  |  |  |  |  |  |
| TG/HDL-C (mean (SD)) | time point 1 | 2.62 (2.09) | 2.60 (2.09) | 2.76 (2.12) | 0.108 |  |
|  | time point 2 | 2.67 (1.76) | 2.65 (1.76) | 2.78 (1.73) | 0.116 |  |
| MetS-IR (mean (SD)) | time point 1 | 34.50 (7.01) | 34.33 (6.94) | 35.61 (7.39) | <0.001 |  |
|  | time point 2 | 34.64 (6.56) | 34.50 (6.53) | 35.65 (6.70) | <0.001 |  |
| TyG-WHtR (mean (SD)) | time point 1 | 4.56 (0.80) | 4.53 (0.78) | 4.71 (0.86) | <0.001 |  |
|  | time point 2 | 4.61 (0.88) | 4.59 (0.89) | 4.81 (0.84) | <0.001 |  |
| SPISE (mean (SD)) | time point 1 | 7.65 (2.04) | 7.70 (2.04) | 7.35 (2.02) | <0.001 |  |
|  | time point 2 | 7.39 (1.95) | 7.43 (1.95) | 7.10 (1.90) | <0.001 |  |
| Note: 1) time point 1 refers to the data in year 2011 in CHARLS database, while time point 2 refers to the data in year 2015 in CHARLS database | | | | | |  |
| 2) MetS-IR refers to metabolic score for insulin resistance, SPISE refers to single-point insulin sensitivity estimator, TG/HDL-C refers to triglycerides to HDL-C ratio, TyG-WHtR refers to TyG waist circumference-to-height ratio. | | | | | |  |

| Table S11 Comparison of the effects of IR trajectories on CVD risk using various markers as exposures | | | | | | |  |
| --- | --- | --- | --- | --- | --- | --- | --- |
| Models | Trajectories | Exposure | Estimate_OR | Std.dev_OR | Pvalue_OR | CI_OR_lower | CI_OR_upper |
| Model1 | Decreasing | MetS-IR | 1.306 | 0.178 | 1.33E-01 | 0.922 | 1.852 |
|  |  | SPISE | 0.869 | 0.169 | 4.06E-01 | 0.624 | 1.210 |
|  |  | TG/HDL-C | 1.127 | 0.154 | 4.38E-01 | 0.833 | 1.523 |
|  |  | TyG-BMI | 1.419 | 0.207 | 9.02E-02 | 0.947 | 2.127 |
|  |  | TyG index | 1.059 | 0.157 | 7.13E-01 | 0.779 | 1.440 |
|  |  | TyG-WHtR | 1.229 | 0.176 | 2.42E-01 | 0.870 | 1.737 |
|  | High stable | MetS-IR | 1.473 | 0.109 | ***3.94E-04*** | 1.189 | 1.825 |
|  |  | SPISE | 0.582 | 0.112 | ***1.32E-06*** | 0.467 | 0.725 |
|  |  | TG/HDL-C | 1.477 | 0.114 | ***6.52E-04*** | 1.180 | 1.848 |
|  |  | TyG-BMI | 1.694 | 0.112 | ***2.32E-06*** | 1.361 | 2.108 |
|  |  | TyG index | 1.459 | 0.117 | ***1.26E-03*** | 1.160 | 1.836 |
|  |  | TyG-WHtR | 1.735 | 0.118 | ***3.00E-06*** | 1.377 | 2.186 |
|  | Increasing | MetS-IR | 1.334 | 0.178 | 1.05E-01 | 0.941 | 1.890 |
|  |  | SPISE | 0.893 | 0.166 | 4.97E-01 | 0.645 | 1.238 |
|  |  | TG/HDL-C | 1.149 | 0.153 | 3.65E-01 | 0.851 | 1.551 |
|  |  | TyG-BMI | 1.527 | 0.159 | ***7.66E-03*** | 1.119 | 2.084 |
|  |  | TyG index | 1.339 | 0.144 | ***4.20E-02*** | 1.011 | 1.775 |
|  |  | TyG-WHtR | 1.597 | 0.168 | ***5.21E-03*** | 1.150 | 2.219 |
| Model2 | Decreasing | MetS-IR | 1.369 | 0.178 | 7.81E-02 | 0.965 | 1.941 |
|  |  | SPISE | 0.880 | 0.170 | 4.49E-01 | 0.631 | 1.226 |
|  |  | TG/HDL-C | 1.142 | 0.154 | 3.91E-01 | 0.844 | 1.546 |
|  |  | TyG-BMI | 1.497 | 0.208 | 5.27E-02 | 0.995 | 2.251 |
|  |  | TyG index | 1.063 | 0.156 | 6.96E-01 | 0.782 | 1.445 |
|  |  | TyG-WHtR | 1.241 | 0.178 | 2.24E-01 | 0.876 | 1.757 |
|  | High stable | MetS-IR | 1.481 | 0.112 | ***4.55E-04*** | 1.189 | 1.844 |
|  |  | SPISE | 0.578 | 0.115 | ***1.77E-06*** | 0.462 | 0.724 |
|  |  | TG/HDL-C | 1.473 | 0.116 | ***8.27E-04*** | 1.174 | 1.848 |
|  |  | TyG-BMI | 1.708 | 0.114 | ***2.51E-06*** | 1.367 | 2.134 |
|  |  | TyG index | 1.441 | 0.118 | ***1.95E-03*** | 1.144 | 1.817 |
|  |  | TyG-WHtR | 1.739 | 0.119 | ***3.40E-06*** | 1.377 | 2.197 |
|  | Increasing | MetS-IR | 1.341 | 0.180 | 1.03E-01 | 0.943 | 1.907 |
|  |  | SPISE | 0.915 | 0.166 | 5.94E-01 | 0.660 | 1.268 |
|  |  | TG/HDL-C | 1.141 | 0.154 | 3.92E-01 | 0.843 | 1.545 |
|  |  | TyG-BMI | 1.537 | 0.161 | ***7.46E-03*** | 1.122 | 2.106 |
|  |  | TyG index | 1.347 | 0.144 | ***3.89E-02*** | 1.015 | 1.788 |
|  |  | TyG-WHtR | 1.609 | 0.168 | ***4.68E-03*** | 1.157 | 2.238 |
| Model3 | Decreasing | MetS-IR | 1.229 | 0.184 | 2.63E-01 | 0.857 | 1.762 |
|  |  | SPISE | 0.989 | 0.172 | 9.49E-01 | 0.706 | 1.386 |
|  |  | TG/HDL-C | 1.114 | 0.155 | 4.85E-01 | 0.823 | 1.508 |
|  |  | TyG-BMI | 1.269 | 0.216 | 2.70E-01 | 0.831 | 1.938 |
|  |  | TyG index | 1.041 | 0.159 | 8.01E-01 | 0.762 | 1.421 |
|  |  | TyG-WHtR | 1.132 | 0.204 | 5.44E-01 | 0.759 | 1.689 |
|  | High stable | MetS-IR | 1.234 | 0.123 | 8.63E-02 | 0.970 | 1.569 |
|  |  | SPISE | 0.679 | 0.127 | ***2.21E-03*** | 0.529 | 0.870 |
|  |  | TG/HDL-C | 1.329 | 0.121 | ***1.87E-02*** | 1.049 | 1.686 |
|  |  | TyG-BMI | 1.389 | 0.127 | ***9.75E-03*** | 1.083 | 1.782 |
|  |  | TyG index | 1.288 | 0.126 | ***4.48E-02*** | 1.006 | 1.649 |
|  |  | TyG-WHtR | 1.374 | 0.158 | ***4.37E-02*** | 1.009 | 1.872 |
|  | Increasing | MetS-IR | 1.294 | 0.186 | 1.66E-01 | 0.899 | 1.862 |
|  |  | SPISE | 0.939 | 0.170 | 7.12E-01 | 0.673 | 1.310 |
|  |  | TG/HDL-C | 1.126 | 0.156 | 4.48E-01 | 0.829 | 1.528 |
|  |  | TyG-BMI | 1.402 | 0.172 | ***4.91E-02*** | 1.001 | 1.962 |
|  |  | TyG index | 1.282 | 0.148 | 9.47E-02 | 0.958 | 1.714 |
|  |  | TyG-WHtR | 1.461 | 0.192 | ***4.82E-02*** | 1.003 | 2.129 |
| Model4 | Decreasing | MetS-IR | 1.362 | 0.267 | 2.47E-01 | 0.807 | 2.296 |
|  |  | SPISE | 1.029 | 0.274 | 9.16E-01 | 0.602 | 1.760 |
|  |  | TG/HDL-C | 1.372 | 0.317 | 3.18E-01 | 0.738 | 2.553 |
|  |  | TyG-BMI | 1.121 | 0.260 | 6.60E-01 | 0.673 | 1.868 |
|  |  | TyG index | 1.110 | 0.275 | 7.04E-01 | 0.648 | 1.902 |
|  |  | TyG-WHtR | 1.374 | 0.204 | 1.19E-01 | 0.922 | 2.048 |
|  | High stable | MetS-IR | 1.042 | 0.194 | 8.33E-01 | 0.713 | 1.523 |
|  |  | SPISE | 0.648 | 0.228 | 5.69E-02 | 0.414 | 1.013 |
|  |  | TG/HDL-C | 1.216 | 0.264 | 4.58E-01 | 0.725 | 2.039 |
|  |  | TyG-BMI | 1.675 | 0.176 | ***3.47E-03*** | 1.185 | 2.366 |
|  |  | TyG index | 1.266 | 0.172 | 1.70E-01 | 0.904 | 1.772 |
|  |  | TyG-WHtR | 1.610 | 0.159 | ***2.78E-03*** | 1.179 | 2.201 |
|  | Increasing | MetS-IR | 1.685 | 0.251 | ***3.75E-02*** | 1.031 | 2.754 |
|  |  | SPISE | 1.099 | 0.318 | 7.66E-01 | 0.589 | 2.051 |
|  |  | TG/HDL-C | 1.075 | 0.271 | 7.90E-01 | 0.632 | 1.826 |
|  |  | TyG-BMI | 1.370 | 0.190 | 9.71E-02 | 0.945 | 1.986 |
|  |  | TyG index | 1.142 | 0.169 | 4.30E-01 | 0.821 | 1.590 |
|  |  | TyG-WHtR | 1.563 | 0.197 | ***2.35E-02*** | 1.062 | 2.301 |
| 1)The effect is estimated from ltmle method with SuperLearner library 1 | | | | |  |  |  |
| 2)SuperLearner library 1 includes generalized linear model in both outcome and exposure modeling in LTMLE. | | | | | | |  |
| 3)Abbreviation: OR, Odds Ratio; CI, Confidence interval. | | | |  |  |  |  |
| 4) Model 1 was adjusted for age and gender; Model 2 further adjusted for model 1 covariates plus residence, marry, education, smoke status and drinking status; Model 3 further adjusted for Model 2 covariates plus comorbidities, including hypertension, dyslipidemia, diabetes, kidney; Model 4 further adjusted for Model 2 covariates plus laboratory parameters, including high-density lipoprotein, low-density lipoprotein, and high-sensitivity C-reactive protein (hsCRP). | | | | | | | |

**Supplementary Figures**


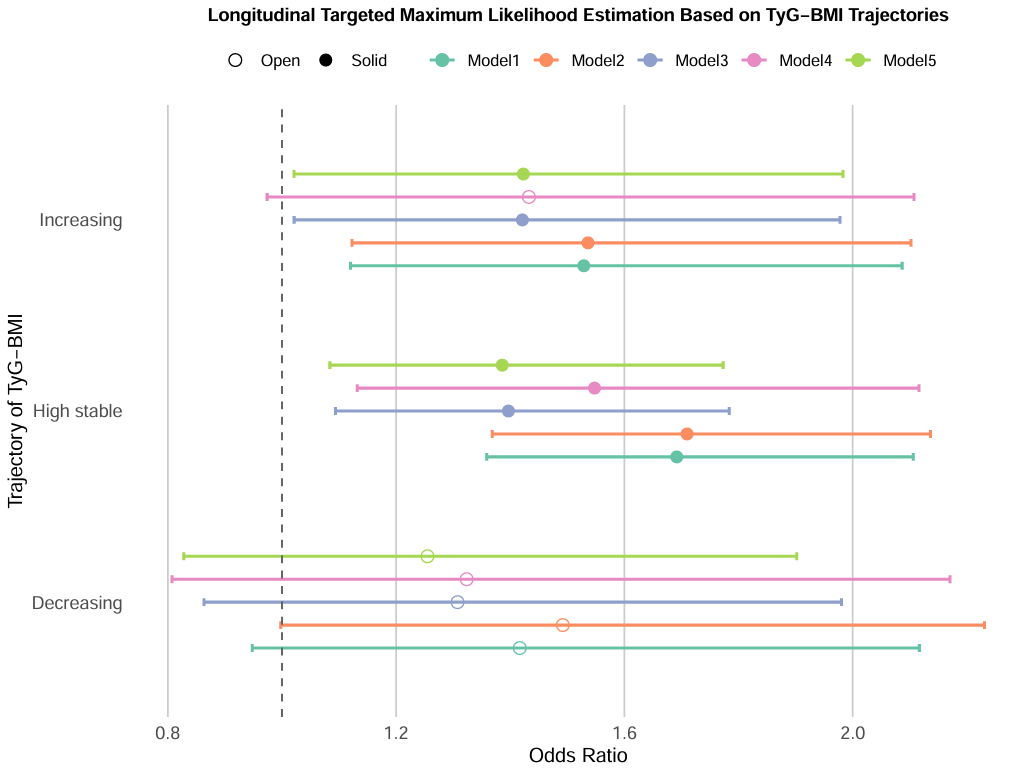


**Fig.S1 Longitudinal targeted maximum likelihood estimation based on TyG-BMI trajectories with SuperLearner library 2**

*1) SuperLearner library 2 includes generalized linear model, mean estimation, lasso and elastic-net regularized generalized linear models, random forest, and xgboost in both outcome and exposure modeling in LTMLE. 2) An open dot indicates the ‘p-value’ is more than 0.05, while a solid dot indicates the significant result with a ‘p-value’ less than 0.05.


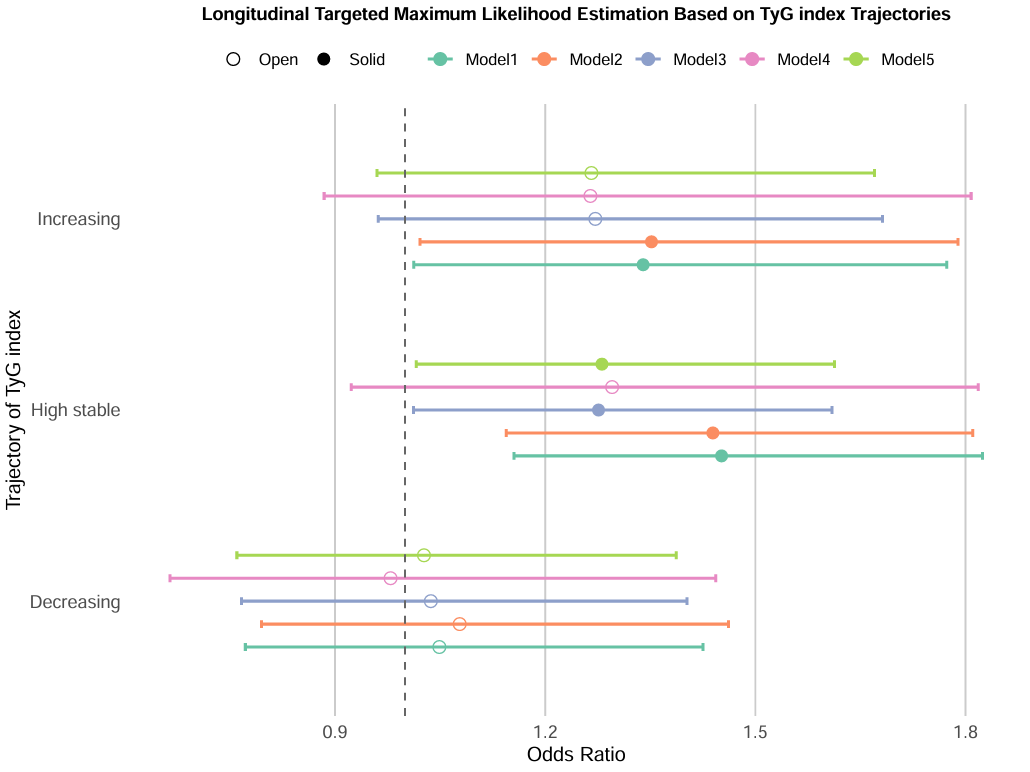


**Fig.S2 Longitudinal targeted maximum likelihood estimation based on TyG index trajectories with SuperLearner library 2**

*1) SuperLearner library 2 includes generalized linear model, mean estimation, lasso and elastic-net regularized generalized linear models, random forest, and xgboost in both outcome and exposure modeling in LTMLE. 2) An open dot indicates the ‘p-value’ is more than 0.05, while a solid dot indicates the significant result with a ‘p-value’ less than 0.05


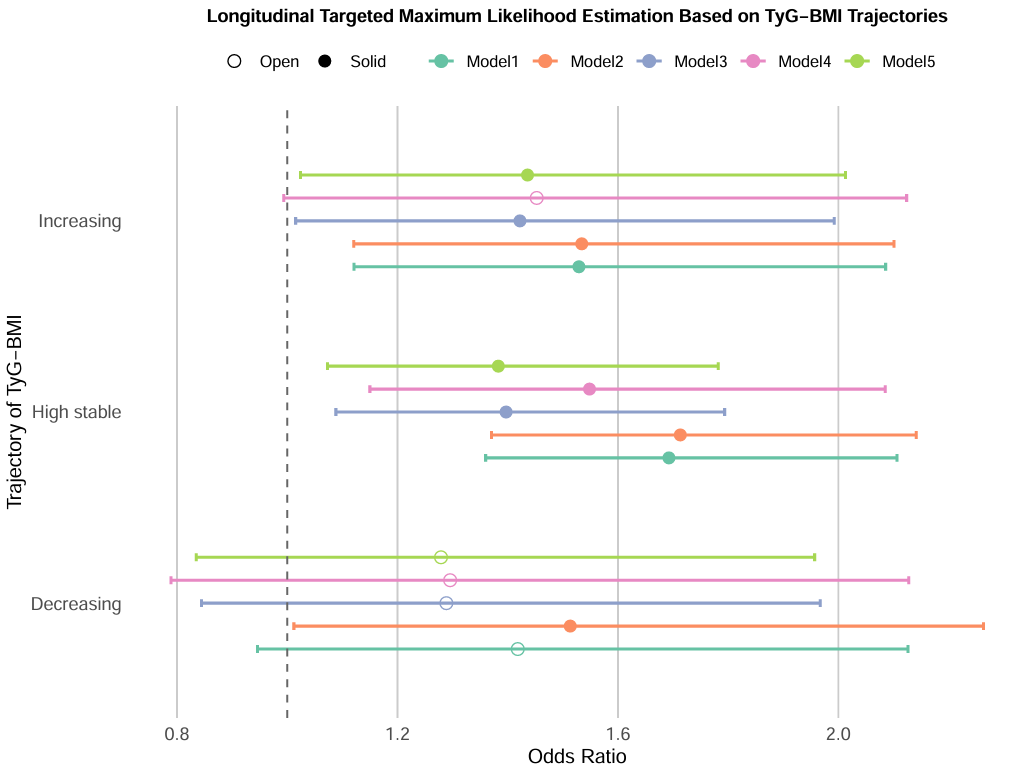


**Fig.S3 Longitudinal targeted maximum likelihood estimation based on TyG-BMI trajectories with SuperLearner library 3**

*1) SuperLearner library 3 includes generalized linear model and xgboost in both outcome and exposure modeling in LTMLE. 2) An open dot indicates the ‘p-value’ is more than 0.05, while a solid dot indicates the significant result with a ‘p-value’ less than 0.05.


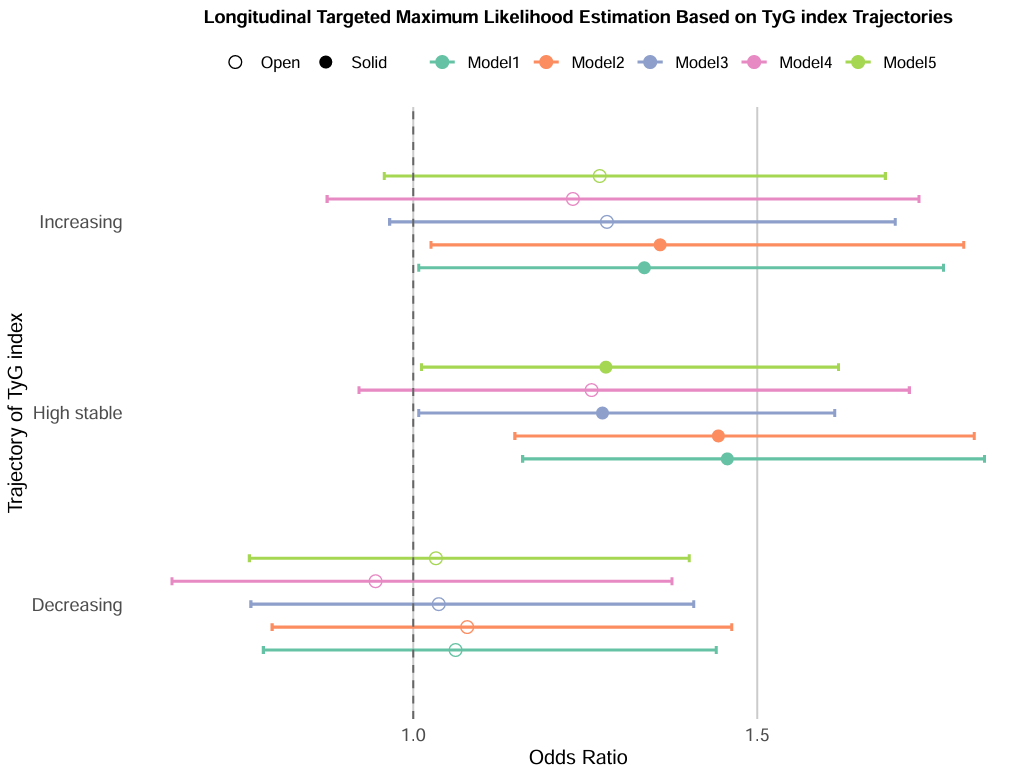


**Fig.S4 Longitudinal targeted maximum likelihood estimation based on TyG index trajectories with SuperLearner library 3**

* 1) SuperLearner library 3 includes generalized linear model and xgboost in both outcome and exposure modeling in LTMLE. 2) An open dot indicates the ‘p-value’ is more than 0.05, while a solid dot indicates the significant result with a ‘p-value’ less than 0.05.


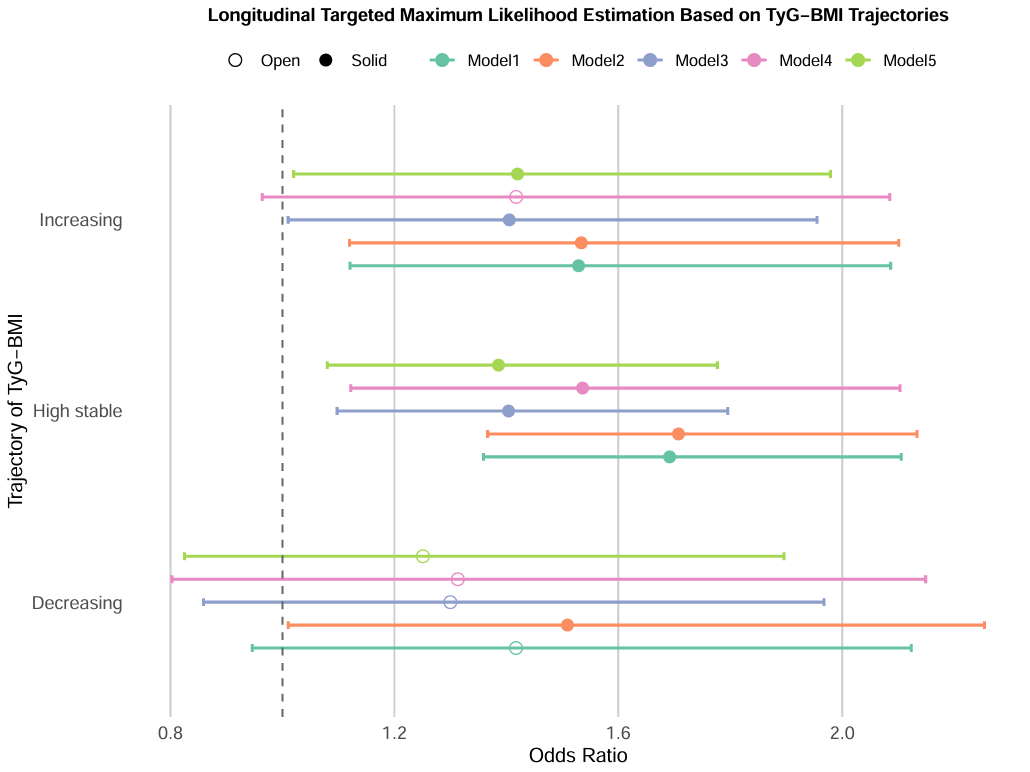


**Fig.S5 Longitudinal targeted maximum likelihood estimation based on TyG-BMI trajectories with SuperLearner library 4**

*1) SuperLearner library 4 includes generalized linear model, mean estimation, random forest, and xgboost in both outcome and exposure modeling in LTMLE. 2) An open dot indicates the ‘p-value’ is more than 0.05, while a solid dot indicates the significant result with a ‘p-value’ less than 0.05.


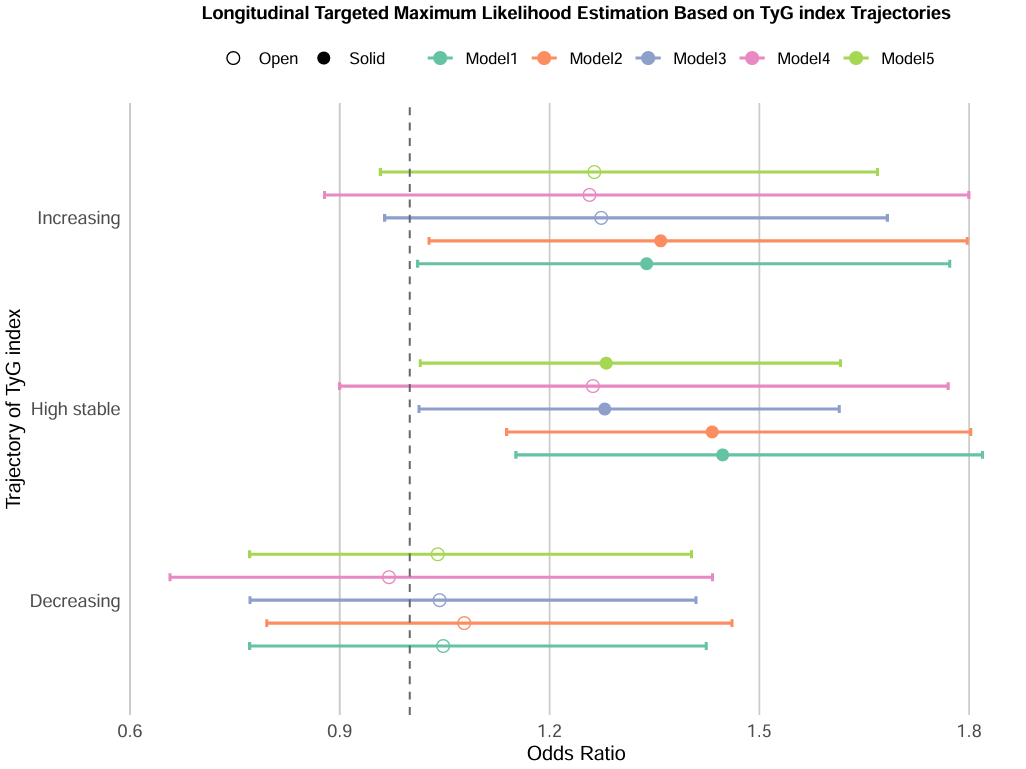


**Fig.S6 Longitudinal targeted maximum likelihood estimation based on TyG index trajectories with SuperLearner library 4**

*1) SuperLearner library 4 includes generalized linear model, mean estimation, random forest, and xgboost in both outcome and exposure modeling in LTMLE. 2) An open dot indicates the ‘p-value’ is more than 0.05, while a solid dot indicates the significant result with a ‘p-value’ less than 0.05.


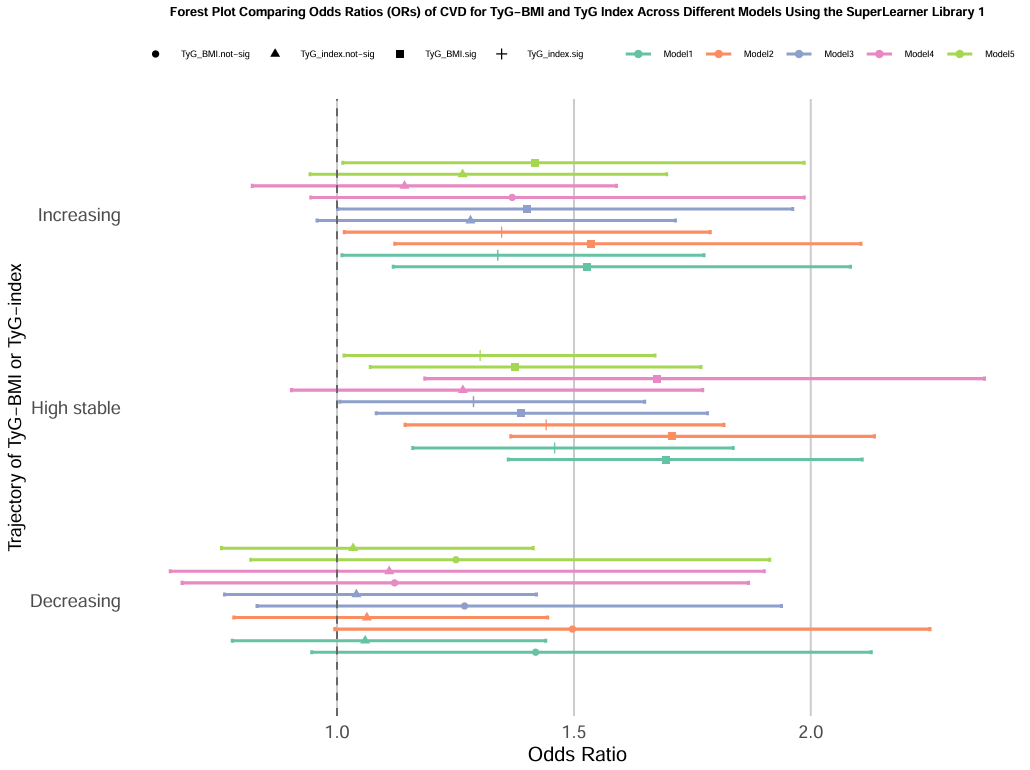


**Fig.S7 Forest plot comparing odds ratios (ORs) of CVD for TyG-BMI and TyG index across different models using the SuperLearner library 1**

*1) SuperLearner library 1 includes generalized linear model. 2) An open dot indicates the ‘p-value’ is more than 0.05, while a solid dot indicates the significant result with a ‘p-value’ less than 0.05.


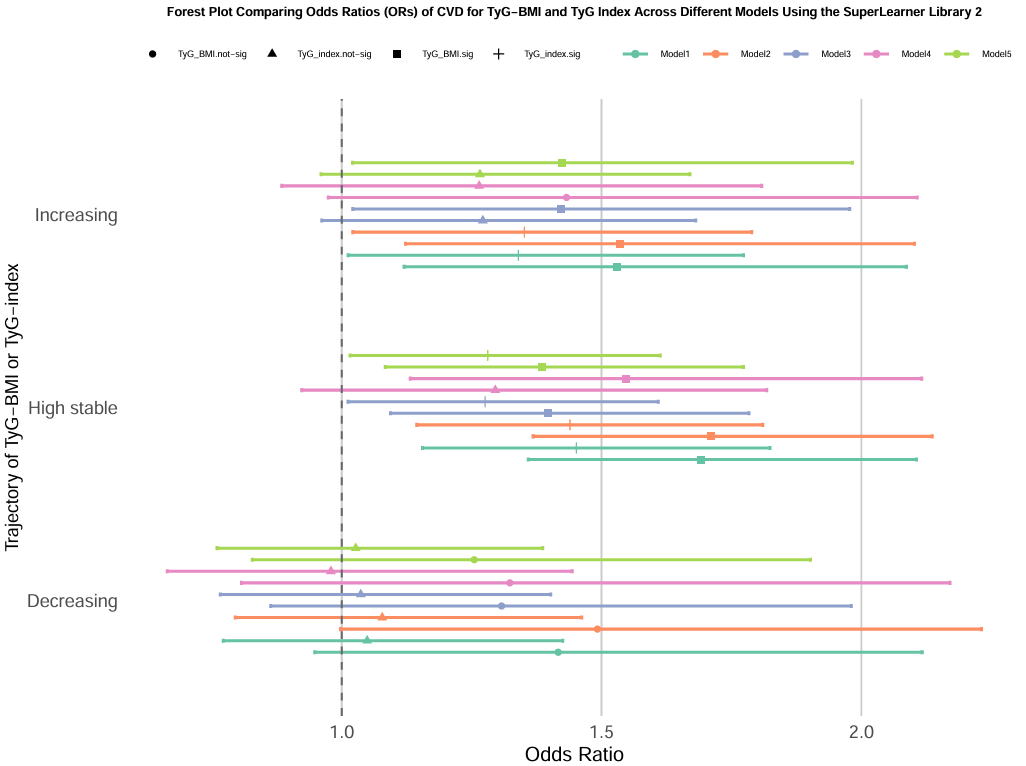


**Fig.S8 Forest plot comparing odds ratios (ORs) of CVD for TyG-BMI and TyG index across different models using the SuperLearner library 2**

* 1) SuperLearner library 2 includes generalized linear model, mean estimation, lasso and elastic-net regularized generalized linear models, random forest, and xgboost in both outcome and exposure modeling in LTMLE. 2) An open dot indicates the ‘p-value’ is more than 0.05, while a solid dot indicates the significant result with a ‘p-value’ less than 0.05.


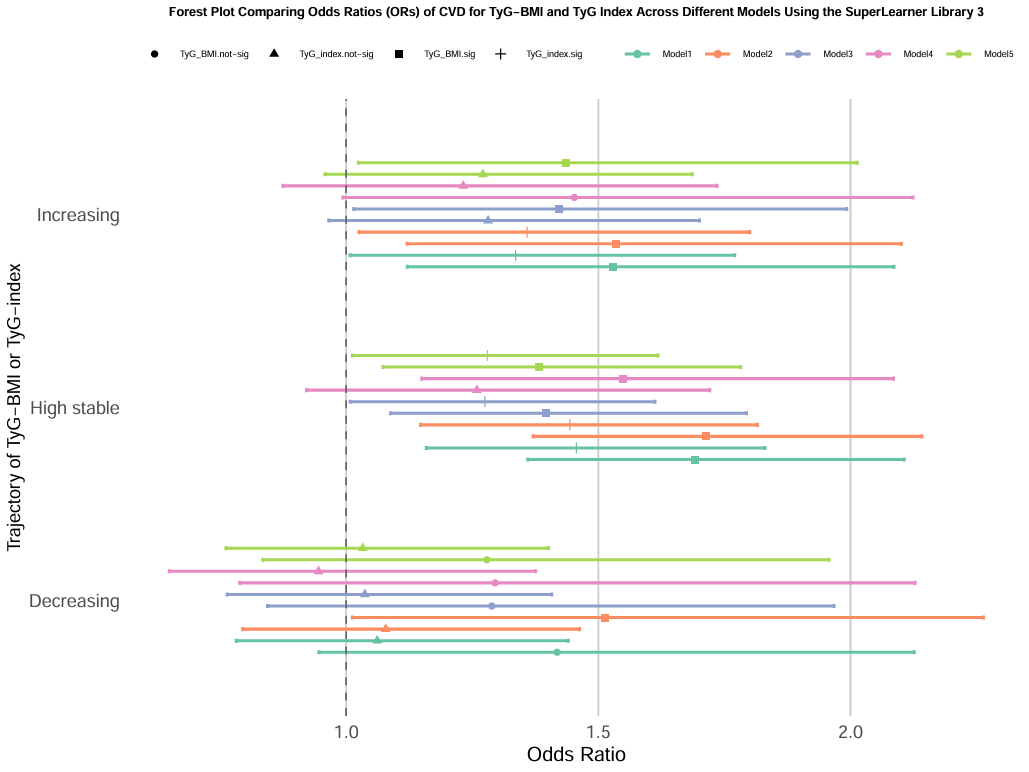


**Fig.S9 Forest plot comparing odds ratios (ORs) of CVD for TyG-BMI and TyG index across different models using the SuperLearner library 3**

*1) SuperLearner library 3 includes generalized linear model and xgboost in both outcome and exposure modeling in LTMLE. 2) An open dot indicates the ‘p-value’ is more than 0.05, while a solid dot indicates the significant result with a ‘p-value’ less than 0.05.


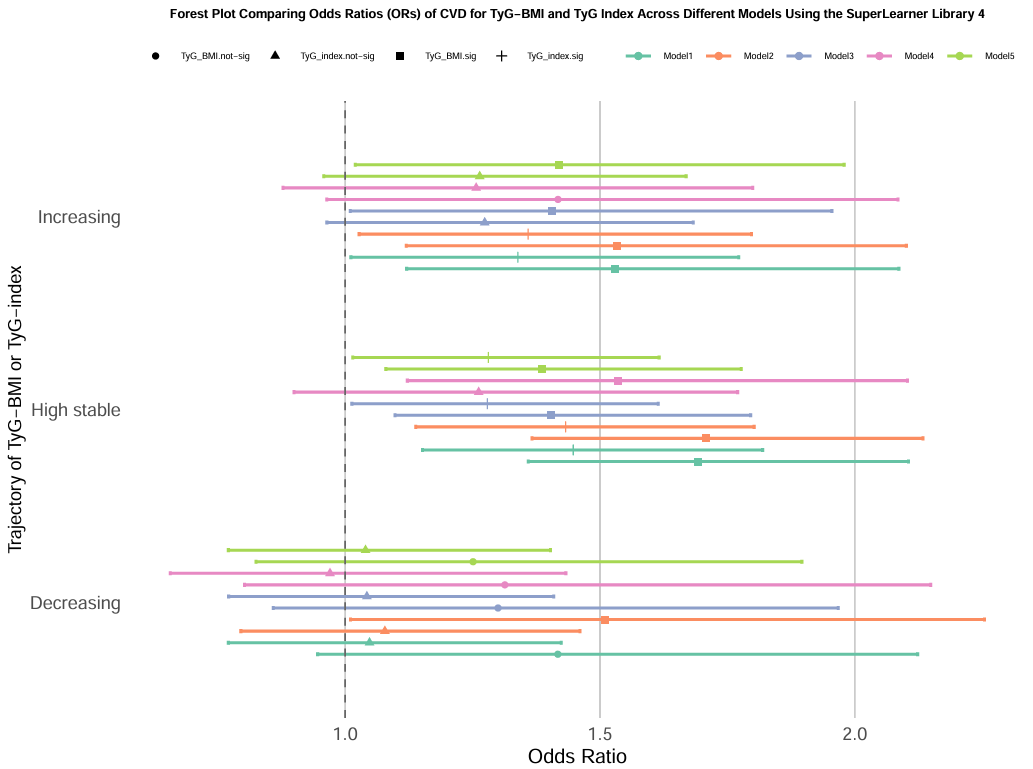


**Fig.S10 Forest plot comparing odds ratios (ORs) of CVD for TyG-BMI and TyG index across different models using the SuperLearner library 4**

* 1) SuperLearner library 4 includes generalized linear model, mean estimation, random forest, and xgboost in both outcome and exposure modeling in LTMLE. 2) An open dot indicates the ‘p-value’ is more than 0.05, while a solid dot indicates the significant result with a ‘p-value’ less than 0.05.
