# Supplementary material for: Assessing the impact of insulin resistance trajectories on cardiovascular disease risk using longitudinal targeted maximum likelihood estimation
Source: Cardiovasc Diabetol. 2025 Mar 10;24:112. doi: 10.1186/s12933-025-02651-6 (PMC11895167; doi:10.1186/s12933-025-02651-6)
Supplement: Supplementary file 2 — Supplementary Material 2 [file 12933_2025_2651_MOESM2_ESM.docx]

## Appendix A Targeted maximum likelihood estimation

Targeted maximum likelihood estimation (TMLE) is a doubly robust method for estimating causal effects^1^. The TMLE considers all the parameters to be nuisance parameters, with the exception of the causal parameter, which is the target of inference^2^. It thus targets its estimation to minimize the bias of that target parameter at the expense of other parameters in the model. TMLE involves estimating an initial conditional expectation $Q_{n}^{0}\left( A,L \right)$of an outcome via maximum likelihood^3^, similar to that created for the G-computation procedure (formula 1), and a conditional expectation of the probability of exposure $g_{n}^{0}\left( A|L \right)$, similar to the propensity score model used by IPW (formula 2). The exposure model is then used to create a ‘clever’ covariate $h$:

$Q_{n}^{0}\left( A,L \right)=E\left( Y|A,L \right)$*, (1)*

$g_{n}^{0}\left( A|L \right)=logit\left( \frac{P\left( A|L \right)}{1-P\left( A|L \right)} \right)$*, (2)*

$h\left( A,L \right)=\frac{I\left( A=1 \right)}{g_{n}^{0}\left( L \right)}-\frac{I\left( A=0 \right)}{g_{n}^{0}\left( L \right)}$*. (3)*

The initial estimate of $E\left( Y|A,L \right)$ is then updated on the basis of a function of the initial estimate and the clever covariate:

$Q_{n}^{*}\left( A,L \right)=Q_{n}^{0}\left( A,L \right)+\epsilon_{n}h\left( A,L \right)$*, (4)*

where $\epsilon_{n}$ is the coefficient of $h\left( A,W \right)$, and the coefficient of $Q_{n}^{0}\left( A,W \right)$ is constrained to one. This can be carried out multiple times, if necessary, and will iterate to convergence (i.e., $\epsilon_{n}=0$), although in the cases of the average treatment effect, only a single iteration is needed^4^, and a single-step TMLE has recently been proposed^5^. The TMLE is then estimated by evaluating the final model at different treatment values:

$\theta_{n}^{TMLE}=\frac{1}{n}\sum_{i=1}^{n} (Q_{n}^{*}\left( 1,L \right)-Q_{n}^{*}\left( 0,L \right))$ *(5)*

This estimator is equivalent to the G-computation estimator, except that the updated $Q_{n}^{*}\left( A,L \right)$ replaces the initial estimate $Q_{n}^{0}\left( A,L \right)$. In other words, the TMLE is similar to the doubly robust method of using an IPTW outcome model to standardize the mean outcome via G-computation. This provides a targeted estimation of the target parameter (the effect of the exposure) at the expense of bias in other parameters in the model.

## Appendix B The assumed causal structure and formula in LTMLE

Below are the examples of the Q and G formulas in the LTMLE model (using the ltmle R package). By default, the models for the dependent variables include all the parent nodes of the preceding time points.

# Define q and g forms for manually specified LTMLE models

Note: *y* refers to the outcome, *l* refers to the confounders, while *a* refers to the exposures.

# Examples of specifying the outcome models

Q.kplus ~ baseline confounders + l_1 + a_1 + l_2 + a_2

# Examples of specifying exposure models

a_1="a_1 ~ baseline confounders + l_1",

a_2="a_2 ~ baseline confounders + l_1 + a_1 + l_2"

## Appendix C Definitions and Specifications of Each Model Used in Our Study

We’ve included different time-varying and time-invariant covariates in our model.

Model 1: CVD ~ age + gender + exposure

Model 2: CVD ~ age + gender + residence + baseline marital status + education + smoke (smoker and non-smoker) + drink + exposure

Model 3: CVD ~ age + gender + residence + baseline marital status + education + smoke (smoker and non-smoker) + drink + Hypertension + Dyslipidemia + Diabetes + Kidney + exposure

Model 4: CVD ~ age + gender + residence + baseline marital status + education + smoke (smoker and non-smoker) + drink + hdl + ldl + crp + exposure

Model 5: CVD ~ age + gender + residence + marital status (time-varying) + education + smoke (ex-smoker, current smoker, and non-smoker) + drink + Hypertension + Dyslipidemia + Diabetes + Kidney + Liver+ exposure

More details of the formula used in LTMLE model.

1. Model 1

# Specifying the outcome models:

Q.kplus1 ~ gender_1M_2F + Age + TyG_BMI_bin + c2015_TyG_BMI_bin

# Specifying the exposure models:

TyG_BMI_bin ~ gender_1M_2F + Age

c2015_TyG_BMI_bin ~ gender_1M_2F + Age + TyG_BMI_bin

1. Model 2

# Specifying the outcome models:

Q.kplus1 ~ gender_1M_2F + residence + marry + education + smoke + drink + Age + c2015_smoke + c2015_drink + c2015_Age + TyG_BMI_bin + c2015_TyG_BMI_bin

# Specifying the exposure models:

TyG_BMI_bin ~ gender_1M_2F + residence + marry + education + smoke + drink + Age + c2015_smoke + c2015_drink + c2015_Age

formula for c2015_TyG_BMI_bin:

c2015_TyG_BMI_bin ~ gender_1M_2F + residence + marry + education + smoke + drink + Age + c2015_smoke + c2015_drink + c2015_Age + TyG_BMI_bin

1. Model 3

# Specifying the outcome models:

Q.kplus1 ~ gender_1M_2F + residence + marry + education + smoke +drink + Age + Hypertension + Dyslipidemia + Diabetes + Kidney + c2015_smoke + c2015_drink + c2015_Age + c2015_Hypertension +c2015_Dyslipidemia + c2015_Diabetes + c2015_Kidney + TyG_BMI_bin +c2015_TyG_BMI_bin

# Specifying the exposure models:

TyG_BMI_bin ~ gender_1M_2F + residence + marry + education + smoke +drink + Age + Hypertension + Dyslipidemia + Diabetes + Kidney +c2015_smoke + c2015_drink + c2015_Age + c2015_Hypertension +c2015_Dyslipidemia + c2015_Diabetes + c2015_Kidney

c2015_TyG_BMI_bin ~ gender_1M_2F + residence + marry + education + smoke + drink + Age + Hypertension + Dyslipidemia + Diabetes + Kidney + c2015_smoke + c2015_drink + c2015_Age + c2015_Hypertension + c2015_Dyslipidemia + c2015_Diabetes + c2015_Kidney + TyG_BMI_bin

1. Model 4

# Specifying the outcome models:

Q.kplus1 ~ gender_1M_2F + residence + marry + education + smoke + drink + Age + hdl + ldl + crp + c2015_smoke + c2015_drink + c2015_Age + c2015_hdl + c2015_ldl + c2015_crp + TyG_BMI_bin + c2015_TyG_BMI_bin

# Specifying the exposure models:

TyG_BMI_bin ~ gender_1M_2F + residence + marry + education + smoke + drink + Age + hdl + ldl + crp + c2015_smoke + c2015_drink + c2015_Age + c2015_hdl + c2015_ldl + c2015_crp

formula for c2015_TyG_BMI_bin:

c2015_TyG_BMI_bin ~ gender_1M_2F + residence + marry + education + smoke + drink + Age + hdl + ldl + crp + c2015_smoke + c2015_drink + c2015_Age + c2015_hdl + c2015_ldl + c2015_crp + TyG_BMI_bin

1. Model 5

# Specifying the outcome models:

Q.kplus1 ~ gender_1M_2F + hukou + education + smoke_new + drink + Age + marry + Hypertension + Dyslipidemia + Diabetes + Kidney + Liver + c2015_smoke_new + c2015_drink + c2015_Age + c2015_marry + c2015_Hypertension + c2015_Dyslipidemia + c2015_Disabetes + c2015_Kidney + c2015_Liver + TyG_BMI_bin + c2015_TyG_BMI_bin

# Specifying the exposure models:

TyG_BMI_bin ~ gender_1M_2F + hukou + education + smoke_new + drink + Age + marry + Hypertension + Dyslipidemia + diabetes + Kidney + Liver + c2015_smoke_new + c2015_drink + c2015_Age + c2015_marry + c2015_Hypertension + c2015_Dyslipidemia + c2015_Disabetes + c2015_Kidney + c2015_Liver

formula for c2015_TyG_BMI_bin:

c2015_TyG_BMI_bin ~ gender_1M_2F + hukou + education + smoke_new + drink + Age + marry + Hypertension + Dyslipidemia + diabetes + Kidney + Liver + c2015_smoke_new + c2015_drink + c2015_Age + c2015_marry + c2015_Hypertension + c2015_Dyslipidemia + c2015_Disabetes + c2015_Kidney + c2015_Liver + TyG_BMI_bin

## Appendix D definition of different SuperLearner library in our study

The SuperLearner^6^ is an ensemble machine learning approach that combines predictions from multiple candidate models to achieve optimal predictive performance. Instead of relying on a single model, SuperLearner uses a weighted combination of various algorithms (e.g., linear regression, random forests, and xgboost, where the weights are determined through cross-validation. This process ensures that the final model capitalizes on the strengths of individual learners while minimizing their weaknesses. We totally defined 4 different SuperLearner libraries in our study. SuperLearner library 1 includes generalized linear model. SuperLearner library 2 includes generalized linear model, mean estimation, lasso and elastic-net regularized generalized linear models, random forest, and xgboost. SuperLearner library 3 includes generalized linear model and xgboost. SuperLearner library 4 includes generalized linear model, mean estimation, random forest, and xgboost^7^.

## Appendix E definition of additional exposures

In our study, additional for IR markers were used for sensitivity analysis, including metabolic score for insulin resistance (MetS-IR), single-point insulin sensitivity estimator (SPISE), triglycerides to HDL-C ratio (TG/HDL-C), TyG waist circumference-to-height ratio (TyG-WHtR)^8-14^. They were calculated by the following formulas:

1. MetS-IR calculation: Ln (2 × fasting glucose [mg/dL] + triglycerides [mg/dL]) × BMI (kg/m^2^)/Ln (HDL-C [mg/dL])
2. SPISE calculation: 600 × HDL-C^0.185^ (mg/dL)/ (triglycerides^0.2^ [mg/dL] × BMI^1.338^ [kg/m^2^])
3. TG/HDL-C calculation: triglycerides (mg/dL)/HDL-C (mg/dL)
4. TyG-WHtR calculation: waist circumference (cm)/height (cm) × Ln (triglycerides [mg/dL] × fasting glucose [mg/dL]/2)

**References**

1. Van Der Laan MJ, Rubin D. Targeted maximum likelihood learning. *The international journal of biostatistics*. 2006;2(1).

2. Bembom O, Petersen ML, Rhee S, et al. Biomarker discovery using targeted maximum‐likelihood estimation: Application to the treatment of antiretroviral‐resistant HIV infection. *Stat Med*. 2009;28(1):152–172.

3. Arnold B, Arana B, Mäusezahl D, Hubbard A, Colford Jr JM. Evaluation of a pre-existing, 3-year household water treatment and handwashing intervention in rural guatemala. *Int J Epidemiol*. 2009;38(6):1651–1661.

4. Gruber S, van der Laan M. Tmle: An R package for targeted maximum likelihood estimation. *Journal of Statistical Software*. 2012;51:1–35.

5. van der Laan M, Gruber S. One-step targeted minimum loss-based estimation based on universal least favorable one-dimensional submodels. *The international journal of biostatistics*. 2016;12(1):351–378.

6. Polley EC, Van der Laan MJ. Super learner in prediction. . 2010.

7. Polley E, LeDell E, Kennedy C, Lendle S, van der Laan M. Package ‘SuperLearner’. *CRAN*. 2019.

8. Qian T, Sheng X, Shen P, Fang Y, Deng Y, Zou G. Mets-IR as a predictor of cardiovascular events in the middle-aged and elderly population and mediator role of blood lipids. *Frontiers in Endocrinology*. 2023;14:1224967.

9. Su X, Zhao C, Zhang X. Association between METS-IR and heart failure: A cross-sectional study. *Frontiers in Endocrinology*. 2024;15:1416462.

10. Deng S, Hu X, Zhang X. Association of single‐point insulin sensitivity estimator index (SPISE) with future cardiovascular outcomes in patients with type 2 diabetes. *Diabetes, Obesity and Metabolism*. 2024.

11. Cederholm J, Zethelius B. SPISE and other fasting indexes of insulin resistance: Risks of coronary heart disease or type 2 diabetes. comparative cross-sectional and longitudinal aspects. *Ups J Med Sci*. 2019;124(4):265–272.

12. He J, He S, Liu K, Wang Y, Shi D, Chen X. The TG/HDL‐C ratio might be a surrogate for insulin resistance in chinese nonobese women. *International Journal of Endocrinology*. 2014;2014(1):105168.

13. McLaughlin T, Abbasi F, Cheal K, Chu J, Lamendola C, Reaven G. Use of metabolic markers to identify overweight individuals who are insulin resistant. *Ann Intern Med*. 2003;139(10):802–809.

14. Kuo T, Lu Y, Yang C, Wang B, Chen L, Su C. Association of insulin resistance indicators with hepatic steatosis and fibrosis in patients with metabolic syndrome. *BMC gastroenterology*. 2024;24(1):26.
